# Chromatin architecture transitions from zebrafish sperm through early embryogenesis

**DOI:** 10.1101/gr.269860.120

**Published:** 2021-06

**Authors:** Candice L. Wike, Yixuan Guo, Mengyao Tan, Ryohei Nakamura, Dana Klatt Shaw, Noelia Díaz, Aneasha F. Whittaker-Tademy, Neva C. Durand, Erez Lieberman Aiden, Juan M. Vaquerizas, David Grunwald, Hiroyuki Takeda, Bradley R. Cairns

**Affiliations:** 1 Howard Hughes Medical Institute, Department of Oncological Sciences and Huntsman Cancer Institute, University of Utah School of Medicine, Salt Lake City, Utah 84112, USA;; 2 Department of Biological Sciences, Graduate School of Science, The University of Tokyo, Tokyo 113-0033, Japan;; 3 Department of Human Genetics, University of Utah, Salt Lake City, Utah 84112, USA;; 4 Max Planck Institute for Molecular Biomedicine, 48149 Muenster, Germany;; 5 The Center for Genome Architecture, Baylor College of Medicine, Houston, Texas 77030, USA;; 6 Department of Molecular and Human Genetics, Baylor College of Medicine, Houston, Texas 77030, USA;; 7 Department of Computer Science, Department of Computational and Applied Mathematics, Rice University, Houston, Texas 77005, USA;; 8 Center for Theoretical Biological Physics, Rice University, Houston, Texas 77030, USA;; 9 MRC London Institute of Medical Sciences, London W12 0NN, United Kingdom;; 10 Institute of Clinical Sciences, Faculty of Medicine, Imperial College London, London W12 0NN, United Kingdom

## Abstract

Chromatin architecture mapping in 3D formats has increased our understanding of how regulatory sequences and gene expression are connected and regulated in a genome. The 3D chromatin genome shows extensive remodeling during embryonic development, and although the cleavage-stage embryos of most species lack structure before zygotic genome activation (pre-ZGA), zebrafish has been reported to have structure. Here, we aimed to determine the chromosomal architecture in paternal/sperm zebrafish gamete cells to discern whether it either resembles or informs early pre-ZGA zebrafish embryo chromatin architecture. First, we assessed the higher-order architecture through advanced low-cell in situ Hi-C. The structure of zebrafish sperm, packaged by histones, lacks topological associated domains and instead displays “hinge-like” domains of ∼150 kb that repeat every 1–2 Mbs, suggesting a condensed repeating structure resembling mitotic chromosomes. The pre-ZGA embryos lacked chromosomal structure, in contrast to prior work, and only developed structure post-ZGA. During post-ZGA, we find chromatin architecture beginning to form at small contact domains of a median length of ∼90 kb. These small contact domains are established at enhancers, including super-enhancers, and chemical inhibition of Ep300a (p300) and Crebbpa (CBP) activity, lowering histone H3K27ac, but not transcription inhibition, diminishes these contacts. Together, this study reveals hinge-like domains in histone-packaged zebrafish sperm chromatin and determines that the initial formation of high-order chromatin architecture in zebrafish embryos occurs after ZGA primarily at enhancers bearing high H3K27ac.

The folding of chromatin inside the nucleus helps regulate enhancer–promoter interactions and the formation of chromatin compartments, which impacts gene regulation and development. Chromatin is organized at multiple scales, the largest of which involves megabase-scale active or inactive regions called A or B compartments, respectively (Lieberman-Aiden et al. 2009). It is further organized into topological associating domains (TADs) that provide a structural framework that enables proper enhancer–promoter loop engagement to minimize improper interactions (Lieberman-Aiden et al. 2009; Dixon et al. 2012; Nora et al. 2012). The disruption of TAD boundaries can misregulate these properties and lead to developmental disorders and promote cancer, demonstrating that TADs are required for proper transcription during development (Gröschel et al. 2014; Northcott et al. 2014; Lupiáñez et al. 2015; Valton and Dekker 2016; Rosa-Garrido et al. 2017; Davis et al. 2018).

A key issue within developmental biology involves how embryos transition from a totipotent to a lineage-committed state, and higher-order chromatin structures are known to influence enhancer–promoter interaction potential for developmental genes. To better understand, we sought to determine how higher-order chromatin structure is initially established in embryos, how they change during early development and cell differentiation, and how they are regulated. Notably, chromatin structure and transcription influence each other, highlighting the need to understand the relationship between the onset of transcription in the embryo (termed zygotic genome activation [ZGA]) and the establishment of the chromatin organization. These issues have been explored in a number of vertebrate and invertebrate species, which have generally revealed that chromatin lacks extensive higher-order structure before ZGA, can form independently of transcription, and largely forms after ZGA (Du et al. 2017; Hug et al. 2017; Ke et al. 2017; Chen et al. 2019). Conversely, *Danio rerio* (zebrafish) has been reported to display both A/B compartments and TAD structures in the early cleavage-stage embryo before ZGA (pre-ZGA). Curiously, both A/B compartments and TADs are lost during ZGA (Kaaij et al. 2018). This apparent observation of pre-ZGA structure is not intuitive in light of the 10 rapid cell cycles (∼15 min/cycle) and DNA replication cycles that accompany zebrafish pre-ZGA embryo development. These reported differences between pre-ZGA (structured and ∼15 min cell cycle) and early post-ZGA (not structured and ∼1 h cell cycle) phases in the zebrafish embryo prompted us to further examine whether the pre-ZGA structure resembles—and is possibly informed by—the structures present in the parental (sperm or oocyte) genomes.

Our work here also addresses zebrafish sperm chromatin architecture. In mammalian species, the vast majority of the paternal genome is packaged in protamine (Carrell 2011; Ausió et al. 2014). However, histones remain focally at many promoters and enhancers of housekeeping and developmental genes in both mice and humans (Hammoud et al. 2009; Brykczynska et al. 2010). In counter distinction to most other vertebrate species, zebrafish sperm genomes are packaged entirely by histones rather than protamine proteins (Wu et al. 2011; Zhang et al. 2016; Zhang et al. 2018), but like mammalian sperm, housekeeping and developmental promoters and enhancers in zebrafish sperm lack DNA methylation and contain H3K4me3, H2A.Z/FV, H3K27ac, and (at developmental genes) H3K27me3 (Wu et al. 2011; Murphy et al. 2018; Zhang et al. 2018). Additionally, histone chromatin marks and DNA methylation are reprogrammed during pre-ZGA zebrafish stages, but in an asymmetric manner; the maternal genome is largely reprogrammed to adopt the marking present in the sperm genome (Bernstein et al. 2006; Wu et al. 2011; Potok et al. 2013; Murphy et al. 2018; Zhang et al. 2018). Prior work in mice and the rhesus monkey strongly suggests the presence of higher-order chromatin in mammalian sperm (Battulin et al. 2015; Jung et al. 2017; Wang et al. 2019), although structure is curiously lacking in human sperm (Chen et al. 2019), suggesting variation in mammals. Thus, an analysis of higher-order structure in zebrafish sperm (which lacks protamine) would complement those prior studies and provide an initial view of the higher-order structure of a genome entirely packaged in histones. Furthermore, if the higher-order structure in sperm resembled the pre-ZGA structure, this would raise the possibility that structure in gametes might be inherited (in part) to influence structure in the embryos. Parental contribution might be diluted by subsequent rapid cell cycles of the developing zebrafish embryo to arrive at the lack of structure seen post-ZGA. This precedent, combined with the technical challenges of conducting high-throughput chromosome confirmation capture (Hi-C) on oocytes versus sperm, prompted our initial analysis of the sperm genome and comparison to the pre-ZGA structure.

Our initial goals were to use Hi-C to provide a better understanding of the connections between chromatin architecture and transcription initiation. We aimed to determine the 3D chromatin conformation of histone-packaged zebrafish sperm and to test if that architecture is transmitted to the next generation and either resembles or guides the structure of pre-ZGA zebrafish embryo chromatin. Notably, our characterization of the sperm genome reveals an architectural feature distinct from TADs and distinct from architecture in embryos. Within embryos, our results at pre-ZGA differed greatly from prior work, prompting a detailed Hi-C and ChIP-seq analysis of post-ZGA samples to identify the locations in the genome where chromatin architecture initially forms.

## Results

### High-resolution Hi-C chromatin conformation maps of zebrafish sperm and early embryos

**Figure 1.**
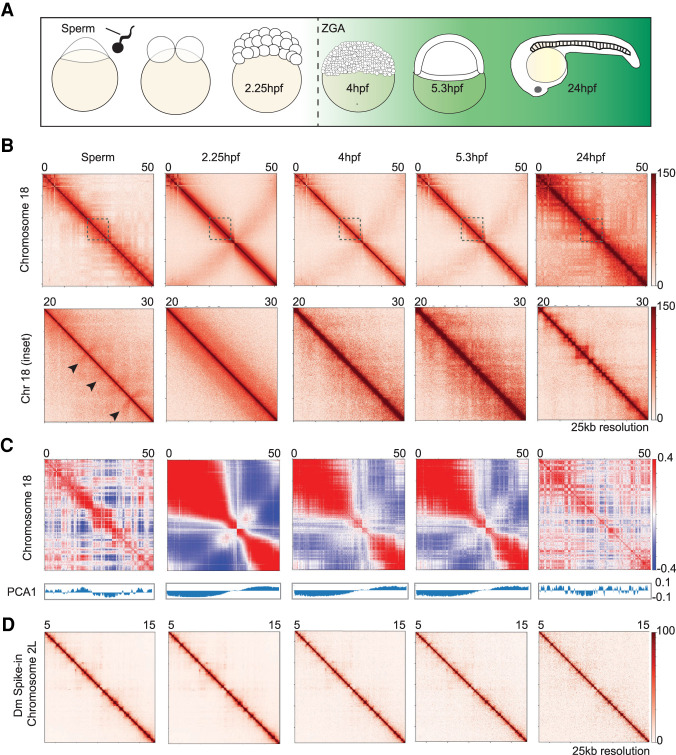
Chromatin architecture in the developing zebrafish sperm and embryos. (*A*) Schematic of the time points collected. Samples collected for low-cell in situ Hi-C experiments: sperm, 2.25 hpf (pre-ZGA, 128-cell), 4 hpf, 5.3 hpf, and 24 hpf. The onset of transcription during zygotic genome activation (ZGA) is ∼3 hpf in zebrafish. The transcription activity is portrayed by the green background. (*B*) Contact matrices of each time point from Chromosome 18 (*top*) inlet is marked in dashed gray box; Chr 18: 20–30 Mb (*bottom*), 25 kb resolution in log scale. Flares detected in sperm time point are marked by black arrows. (*C*) Correlation matrix of each time point from Chr 18. The first eigenvector (PCA1) for the normalized observed/expected ratio is shown *below* the panel to determine A/B compartment status. (*D*) Contact matrices of each time point from the *Drosophila* S2 spike-in Chr 2L: 5–15 Mb, 25 kb resolution in log scale.

To better understand the nucleation of chromatin architecture in the developing embryo, our time course focused on time points that flank and include ZGA. To these ends, we modified previously published low-cell input Hi-C methods to the early zebrafish embryo (Methods; Rao et al. 2014; Díaz et al. 2018) and determined the 3D chromatin organization of zebrafish mature sperm, as well as embryos at 2.25 hpf (pre-ZGA), 4 hpf (just after ZGA initiation), 5.3 hpf (post-ZGA, gastrulation), and 24 hpf (Fig. 1A; Supplemental Fig. S1A). To ensure clear interpretation, we generated Hi-C contact maps of higher resolution than prior work (Kaaij et al. 2018; Supplemental Table S1).

Visual inspection of normalized contact probability maps for all samples at 25 kb resolution revealed considerable differences in structure within the zebrafish developmental stages examined (for Hi-C statistics, see Supplemental Table S1). First, we observed differences in contact probability over genomic distance for each embryo time point (Supplemental Fig. S1B), suggesting that the overall chromatin architecture in the developing zebrafish embryo varies between time points. Consistent with prior work (Kaaij et al. 2018), genomes of 24 hpf embryos show clear 3D structures of traditional triangular topological associated domains (TADs) (Fig. 1B). Sperm chromatin lacked TADs and instead displayed a unique structure, one that resembled “flare-like” structures, in the contact maps that was not observed in embryo stages (Fig. 1B). Regarding the embryo, the contact maps in pre-ZGA (2.25 hpf) lacked TAD-like structure domains, in marked contrast with a prior report (Kaaij et al. 2018). For both post-ZGA samples (4 hpf and 5.3 hpf), only a limited number of regions formed small contact domains, which were detectable by the changes in chromatin interactions along the diagonal that are smaller than a TAD size (Fig. 1B), explored in detail below. Furthermore, the self-interacting A/B chromatin compartments were largely absent in our pre-ZGA through post-ZGA samples, although they were clearly detected in sperm and at 24 hpf (Fig. 1C). Examination of our data by HiCExplorer (Wolff et al. 2018), a program to analyze Hi-C data, revealed a lack of boundary structures in sperm or pre-ZGA samples (Supplemental Fig. S1C). We then generated metaplots of aggregate TAD insulation signal, using boundaries established at 24 hpf, and this approach also showed a lack of negative insulation score in sperm and pre-ZGA samples (Supplemental Fig. S1D). Thus, our initial premise that sperm architecture might resemble the reported structure in pre-ZGA embryos was not supported, prompting instead an exploration of why the pre-ZGA samples lacked both A/B compartments and TAD boundaries, where and when structure initially forms in zebrafish, and the characterization of the unique structures observed in sperm.

### Zebrafish pre-ZGA embryos lack a defined 3D architecture

As previewed above, we found the pre-ZGA (2.25 hpf) genome essentially void of boundaries and TAD-like chromatin interactions (Fig. 1B; Supplemental Fig. S1C). We took two measures to determine whether the observed lack of structure in pre-ZGA samples was biological or instead a technical artifact. First, to minimize the confounding effects of highly condensed mitotic chromatin, we took advantage of the cell cycle synchrony of pre-ZGA embryos to enrich for embryo batches that were largely outside metaphase by including only embryo batches with <30% metaphase contribution in our pre-ZGA samples (Methods; Supplemental Fig. S1E). Second, to address whether the lack of structure during pre-ZGA was technical or biological, we examined Hi-C contact maps and contact probability with genomic distance plots of the *Drosophila* spike-in (Fig. 1D; Supplemental Fig. S1F). For all time points, the spike-in positive controls looked identical, which suggests a biological rather than a technical basis for the absence of structure during pre-ZGA. Taken together, these results suggest that zebrafish early embryos lack a defined 3D architecture. Independent work of our collaborators, working in both *Oryzias latipes* (medaka) and zebrafish, came to similar conclusions (Nakamura et al. 2021).

### Low-cell Hi-C method with pre-ZGA embryos is susceptible to somatic cell contamination

**Figure 2.**
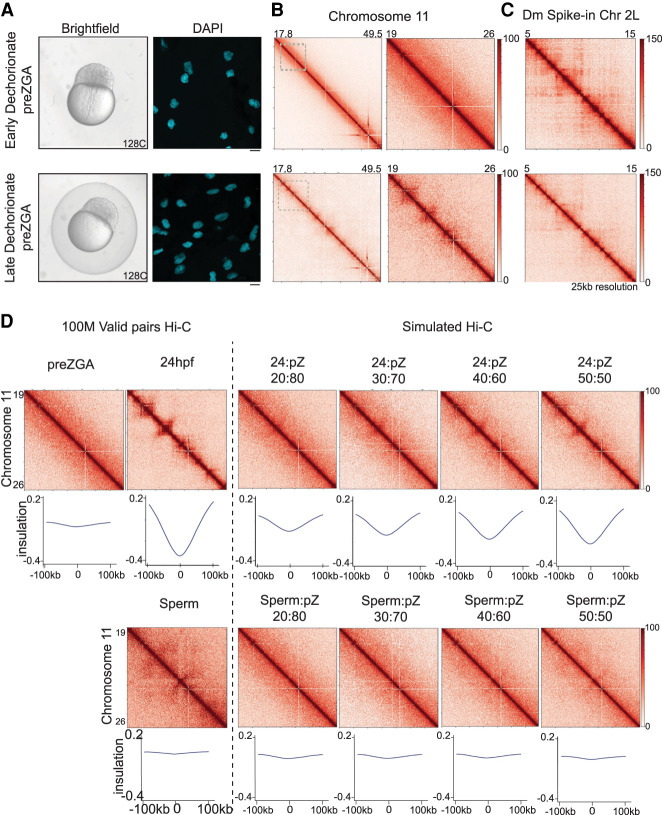
Impact of alternative chorion removal procedures on perceived chromatin architecture. (*A*) Brightfield images of embryos collected at pre-ZGA/128-cell: early dechorionated (at 128-cell stage; *top left*), late dechorionated (just before fixation; *bottom left*). pre-ZGA/128-cell embryo fixed and the DNA was stained with DAPI (cyan) dechorionated at one-cell stage (*top right*) and dechorionated before fixation (*bottom right*) with 40× obj scale bar = 10 µM. (*B*) Contact matrices from pre-ZGA/128-cell, whole Chromosome 11 (early dechorionated, *top left*; late dechorionated, *bottom left*) inlet is marked in dashed gray box; partial Chr 11: 19–26 Mb (early dechorionated, *top right*; late dechorionated, *bottom right*), 25-kb resolution in log scale. (*C*) Contact matrices from the *Drosophila* S2 spike-in, Chr 2L: 5–15 Mb (early dechorionated, *top*; late dechorionated, *bottom*), 25-kb resolution in log scale. (*D*) Contact matrices from Chr 11: 19–26 Mb pre-ZGA/128-cell, 24 hpf, and sperm down-sampled to 100 M valid pairs (*left* of dashed line). Simulated contact matrices (*right* of dashed line) are imaged of pre-ZGA/128-cell with increasing percentages of 24 hpf (*top*) or sperm (*bottom*) valid pairs. Metaplots for the boundaries called at 24 hpf (25-kb resolution) are plotted *below* each matrix.

We then sought to explain how structure might have been observed during the pre-ZGA stage in prior work (Kaaij et al. 2018). A major challenge involves the need to isolate chromatin from embryos that are initially encased in a chorion. During oocyte maturation, the chorion is surrounded by (and in association with) large numbers of somatic granulosa and theca cells, which can remain on the surface of the chorion during early embryo stages and must be properly removed (Selman et al. 1993). We found a significant difference depending on whether the chorion was removed immediately before embryo fixation (late dechorionation) versus at the one-cell stage shortly after fertilization (early dechorionation). Although late dechorionated pre-ZGA embryos showed chromatin contacts that strongly resemble prior work, early dechorionated pre-ZGA embryos lacked 3D conformation features (Fig. 2A–C). This suggests that contamination is likely responsible for the pre-ZGA structural features reported previously.

To determine the source of contamination, we examined whether the structured contact maps from late dechorionation better resembled maps from sperm chromatin or somatic cell chromatin. We simulated Hi-C contact maps by mixing pre-ZGA valid pairs with increasing percentage of valid pairs from 24 hpf or sperm data sets (Fig. 2D) and then generated metaplots of aggregate TAD insulation signal, using boundaries established at 24 hpf (Fig. 2D). Structure was detectable genome-wide with 30% mixing of 24 hpf valid pairs; because the 2.25 hpf embryo has only 128 cells, this level/percentage of contamination might easily be reached. These analyses suggest that the structure detected in the pre-ZGA embryo (Fig. 2B) with late dechorionation involves somatic cell contamination, and not sperm contamination. Here, we speculate that the late removal of the chorion from pre-ZGA samples causes shedding of somatic cells from the chorion surface, and subsequent reassociation with the exposed/dechrorionated embryos, to provide a source of contamination.

### Boundaries established at 4 hpf are maintained through development

We then used our high-resolution contact maps to explore where embryonic chromosomal structures are initially formed during and/or after ZGA (Fig. 1A; Supplemental Table S1). First, 5.3 hpf (post-ZGA) and 24 hpf stages displayed chromatin architecture interactions at similar locations, although stronger at 24 hpf than at 5.3 hpf, a trend noted in previous work and confirmed through our reanalysis of that prior data (Supplemental Fig. S2; Kaaij et al. 2018). Our analysis also validates prior observations that the 4, 5.3, and 24 hpf staged embryos progressively form chromatin interactions and TADs during development (Supplemental Fig. S2A). However, examination of our data and prior data revealed chromosomal domains and boundary-like structures at 4 hpf that were not detected in prior work and a higher overlap between time points (Supplemental Fig. S2B–D). By measuring negative insulation scores across each time point, we observed a limited number of structural boundaries emerging at 4 hpf, and that those established at 4 hpf are largely maintained at 5.3 and 24 hpf (Supplemental Fig. S2E,F). The clarity of TADs in 24 hpf embryos, which are diverse in cell types, shows that zebrafish share with other species a consistency in TAD organization between cell types (Dixon et al. 2012, 2015; Vietri Rudan et al. 2015; Hug et al. 2017).

### Chromatin architecture boundaries persist in the absence of transcription

We next investigated the relationship of TAD boundaries to transcription. We first determined the association of RNA Polymerase II (Pol II) during TAD boundary establishment by evaluating the insulation score across the top 1000 peaks of Pol II loci ChIP-seq at 4 hpf embryos. Pol II–bound loci at 4 hpf displayed negative insulation scores, suggesting that Pol II–bound loci are associated with TAD boundaries forming/formed at 4 hpf (Supplemental Fig. S3A). We next addressed whether the loss of productive Pol II elongation at these boundaries impacts chromatin organization. To test, we treated zebrafish embryos with either vehicle (DMSO) or the Pol II inhibitor Flavopiridol (FLAV), starting at the one-cell stage and continuing through ZGA, and collected embryos at 4 hpf for examination by in situ Hi-C (Supplemental Fig. S3A,B; Supplemental Table S1). Treatment with FLAV led to a loss of Pol II ser5 phosphorylation signal, by immunofluorescence, suggesting effective Pol II inhibition and loss of transcription in the 4 hpf embryo (Supplemental Fig. S3C). However, the chromatin Hi-C contact maps obtained after Pol II inhibition appeared largely unaffected (Supplemental Fig. S3B). TAD boundary insulation present at Pol II–bound loci in the 4 hpf (untreated) and vehicle-treated (DMSO) embryos was only slightly reduced upon treatment with FLAV. These results suggest that the lack of transcription elongation is not sufficient to markedly disrupt chromatin architecture boundaries, a result supported by similar experiments (Hug et al. 2017; Ke et al. 2017; Kaaij et al. 2018).

### Chromatin boundaries correlate with predicted Ctcf sites, whereas Rad21/cohesin-occupied regions have small contact domains

Although TAD structures are rare and weak at ZGA, we sought to address instead whether smaller contact domains might be established in early zebrafish embryos, and by virtue of their small size, form in spite of replication/cell cycle time constraints. Prior work in other systems suggested that early enhancer/promoter loops might form independent of cohesin and Ctcf (CTCF ortholog) co-occupied sites and can form faster than structural loops (Zhang et al. 2019), prompting an examination of zebrafish Ctcf binding sites, cohesin, and enhancers at 4 hpf. Here, anti-Rad21 antibodies are available, whereas commercial zebrafish anti-Ctcf antibodies are not available, requiring instead our procuring potential Ctcf binding sites by HOMER Motif finder across the Zv10 genome. We verified the presence of RNAs during ZGA encoding structural proteins (Ctcf and cohesin complex) and cohesin loading and unloading factors (Nipbl and Wapl) using publicly available RNA-seq data (Supplemental Fig. S4A,B; White et al. 2017). At post-ZGA (4 hpf), the locations where both Rad21 (cohesin; via ChIP-seq) and candidate Ctcf binding sites were coincident, we also observe chromatin architecture boundaries (a decrease in insulation score, as depicted by blue signal in the heatmap) across all developmental time points (Supplemental Fig. S4C). In contrast, at locations where Rad21 binds independent of the presence of Ctcf binding sites, the opposite behavior was observed—an increase in interactions (increase in insulation score, as depicted by red signal in the heatmap), especially at 5.3 hpf (Supplemental Fig. S4C). These observations suggest that small contact domains (median size 90 kb) occur in the early embryo at locations where cohesin is present, but not where Ctcf is predicted to be co-occupied with cohesin.

### Chromatin architecture is initially established at putative ZGA enhancers

**Figure 3.**
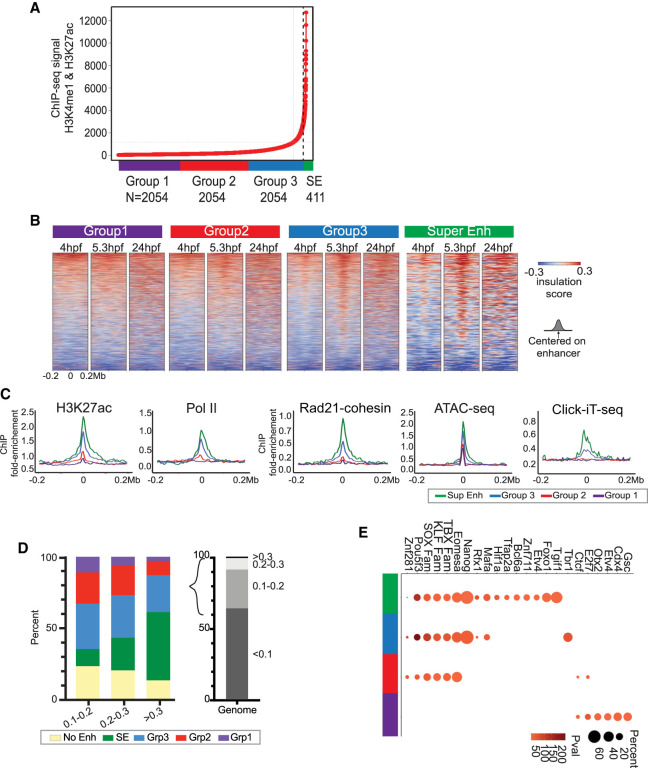
Characterization of chromatin architecture established at enhancers and super-enhancers at 4 hpf. (*A*) Super-enhancer (SE) plot using the ROSE algorithm, which ranks enhancers based on histone H3K27ac (Zhang et al. 2018) and H3K4me1 (Bogdanovic et al. 2012). ChIP-seq data at 4 hpf in zebrafish embryos. Data are separated into four groups: Group 1 (purple), Group 2 (red), Group 3 (blue), and SE group (green). *N* = total number in each partition. (*B*) Heatmaps of insulation score at enhancers. Insulation maps at 4, 5.3, and 24 hpf ranked by insulation strength at 4 hpf, centered on enhancers from each respective group in Figure 4A. Positive insulation (red) indicates increased contacts, and negative insulation (blue) indicates a lack of contacts. (*C*) Comparisons of chromatin factor and attribute occupancy at enhancers. Metaplot of log_2_ fold enrichment of histone H3K27ac ChIP-seq (Zhang et al. 2018), RNA Pol II ChIP-seq, Rad21-cohesin ChIP-seq, ATAC-seq, and Click-iT-seq (Chan et al. 2019) signal over input are plotted, centered on enhancers from each respective group in Figure 4A: super-enhancers (Super Enh, green), Group 3 (blue), Group 2 (red), and Group 1 (purple). (*D*) Proportional distribution of different enhancer regions; no enhancer (No Enh, yellow), super-enhancers (SE, green) Group 3 (Grp3, blue), Group 2 (Grp2, red), and Group 1 (Grp1, purple) detected with positive insulation score 0.1–0.2, 0.2–0.3, >0.3. The proportional distribution of each positive insulation score detected over the entire genome is depicted on the *right*; 0–0.1 (63%), 0.1–0.2 (27%), 0.2–0.3 (8%), >0.3 (1%). The bracket highlights the positive insulation score used in the bar graph on the *left*. (*E*) Groups from *A* overlap with ATAC-seq peak signal across enhancers regions were analyzed using HOMER Motif Analysis to determine potential TF binding. Similarity to known binding motifs is indicated by Pearson R values in shaded red, and motif frequency is indicated by circle size. T-box transcription protein family of motifs (TBX Fam), Kruppel-like factor protein family of motifs (KLF FAM), SRY-box transcription factor protein family of motifs (SOX).

We next explored possible chromatin interactions at enhancers and their relationships to cohesin, Ctcf, and other DNA-binding proteins. First, we defined candidate enhancers at 4 hpf using the standard ROSE algorithms (Lovén et al. 2013; Whyte et al. 2013) and published ChIP-seq data sets for H3K27ac and H3K4me1 (Zhang et al. 2016). The top-ranking regions were defined as candidate super-enhancers (SE), and the remaining ranked enhancers were stratified into three equal-sized cohorts for further examination, labeled Groups 1–3 (Fig. 3A). Enhancers with high levels of histone H3K27ac and H3K4me1 displayed positive insulation scores, at 4 and 5.3 hpf (SE and Group 3) suggesting that these putative enhancers are associated with chromatin interactions, with higher insulation scores detected at 5.3 hpf (Fig. 3B). These small contact domains found at strong enhancers have a median length of 90 kb. In contrast, Groups 1 and 2, which displayed relatively low levels of histone H3K27ac and H3K4me3, lacked small contact domains (Fig. 3B).

To determine whether enhancers are the primary location where structure is initially established, we examined all regions of chromosomal interaction/structure (measured by positive insulation score) and determined the proportion of those regions that contain enhancers. First, we captured and stratified regions with positive insulation signal at 5.3 hpf genome-wide and determined the number of enhancer types within each positive insulation region. Enhancers constituted the majority of regions above the threshold positive insulation score 0.1, and the regions with the highest positive insulation scores consisted mainly of Group 3 or SE enhancers (Fig. 3D). Only a small proportion of the enhancers that display structure during ZGA are retained at 24 hpf, suggesting that only a portion of the enhancer repertoire used at ZGA is similarly used at 24 hpf (Supplemental Fig. S5B). Taken together, enhancers constitute the primary regions of the genome where structural interactions initially form, and their insulation score strength scales with their levels of H3K27ac and H3K4me1.

### Small contact domains at ZGA correlate with pluripotency factors and transcription, but interactions do not require active transcription

We next determined whether transcription factors or architectural structural proteins might bind at these enhancer regions to help establish enhancer domains and/or enhancer–promoter loops in the developing embryo, possibly to help prime these loci for future transcription. To test for factor binding, we performed the Assay for Transposase-Accessible Chromatin (ATAC-seq) in the 4 hpf embryo. The ATAC-seq signal had the strongest peak across potential SE and Group 3 enhancers (Fig. 3C; Supplemental Fig. S5A). We then intersected the enhancer regions with the ATAC-seq signal and used HOMER Motif Analysis (Heinz et al. 2010) to determine candidate transcription factors that bind at these putative enhancers at 4 hpf. Additionally, we confirmed whether a candidate binding factor is indeed expressed at the RNA level in the early embryo by cross-referencing with RNA-seq data sets (Chan et al. 2019). This approach yielded sites for several important transcription factors related to pluripotency, for example, Pou5f3-family (POU5F1 [also known as OCT4] human ortholog), Sox-family, and Nanog-family members were more enriched in SE and Group3 relative to the other groups (Fig. 3E). Here, Ctcf motifs only appeared in Group 2 and Group 1 enhancer groups, further supporting that sites of strong interaction lack Ctcf. Overall, multiple transcription factors and structural proteins—especially those associated with regulating pluripotency—appear to have in silico enrichment for their motif across all enhancer groups.

To determine the histone modifications or chromatin features that best correlate with interaction scores across 4 hpf enhancers, we examined published 4 hpf embryo ChIP-seq profiles of histone H3K27ac (Zhang et al. 2018), H3K4me1 (Bogdanovic et al. 2012), H3K4me3 (Zhang et al. 2018), H3K27me3 (Zhang et al. 2018), H3K36me3 (Zhang et al. 2018), and our ChIP-seq data of Pol II, and Rad21 (cohesin) centered at the enhancers (Fig. 3C; Supplemental Fig. S5A). First, histone H3K27ac and H3K4me1 were expectedly coincident, and H3K4me3 and H3K27me3 were low or lacking at the strongest enhancers—those with highest histone H3K4me1 and H3K27ac (SE and Group 3) (Fig. 3C; Supplemental Fig. S5A). Additionally, cohesin, Pol II, and H3K36me3 were detected across regions within SE and Group 3 putative enhancers (Fig. 3C; Supplemental Fig. S5A). Although these heatmaps appear to convey a direct overlap of Pol II and histone H3K36me3 at enhancers, our limited resolution (>10-kb bins) cannot reveal enhancer/promoter looping. Next, to distinguish from maternally deposited mRNAs from actively transcribed mRNAs (and enhancer-derived RNAs [eRNAs]) from the zygotic genome, we analyzed published zebrafish embryo Click-iT-seq (Chan et al. 2019), which revealed that in SE and Group 3 their clear regional coincidence of Pol II and active transcription (Fig. 3C). Taken together, regions that combine high levels of histone H3K27ac and H3K4me1, together with open chromatin (at transcription factor binding sites) and active transcription, display increased chromatin interactions and define an early chromatin architecture specific to the developing embryo.

**Figure 4.**
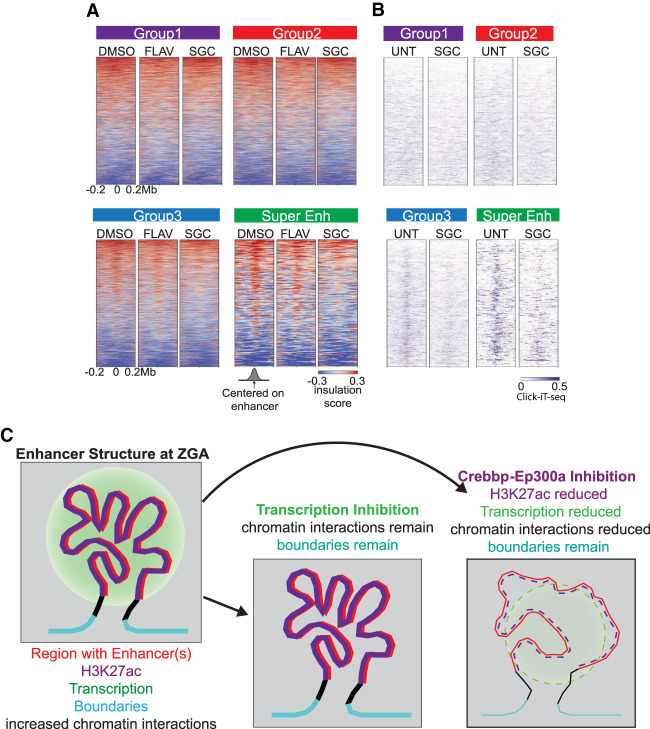
Inhibition of Crebbp/Ep300a causes loss of chromatin architecture around the established super-enhancers at 4 hpf. (*A*) Heatmaps of insulation score for drug-treated embryos. Treatments involve DMSO (vehicle), flavopiridol (FLAV), SGC-CP30 (SGC) for 4 h (which causes a developmental arrest for FLAV and SGC). Respective enhancer groups are ranked as in Figure 3B. Positive insulation (red) indicates increased contacts, and negative insulation (blue) indicates a lack of contacts. (*B*) Click-iT-seq of 4 hpf untreated (UNT) and SGC-CP30 (SGC, purple) (Chan et al. 2019) heatmaps centered on enhancers of each respective enhancer group ranked as in Figure 4A. (*C*) Model depicting the features present at regions displaying structure/positive interaction. Features displayed include enhancers (red), elevated histone H3K27ac (purple), active transcription (green circle), defined boundaries (cyan), and increased chromatin interactions as detected by positive interaction scores in Hi-C contact maps at both 4 and 5.3 hpf. Regions with increased interactions are typically coated with histone H3K27ac. These interactions and boundaries persist upon inhibition of RNA Pol II initiation at 4 hpf. In contrast, these contacts between boundaries are lost upon inhibition of Crebbp/Ep300a (lowering histone H3K27ac [dashed]) leading to decreased transcription and loss of higher-order chromatin structure; however, boundaries remain stable.

We have shown that loss of transcription at boundaries had only minor effects on TAD-scale chromatin architecture. To test whether these small contact domains at putative enhancers relies on Pol II activity, we analyzed our Hi-C contact maps of Pol II-inhibited samples (the aforementioned FLAV treatment) for chromatin insulation score, centered on enhancer regions (Fig. 4A). Again, we observed little to no impact on chromatin insulation score following Pol II inhibition, confirming that chromatin architecture, boundary, and the small contact domain establishment is also largely independent of Pol II transcription (Hug et al. 2017; Ke et al. 2017).

### Crebbp/Ep300a activity helps establish chromatin interactions at enhancers

Because active Pol II transcription itself is not required for the formation of chromatin architecture, we asked instead whether transcription-independent histone post-translational modifications placed on enhancers might help establish chromatin architecture in the early embryo. Prior work in cell culture has shown that regions with high histone H3K27ac are able to form small contact domains, which are established faster and often independent of CTCF/cohesin co-occupying sites (Rao et al. 2017; Zhang et al. 2019). Therefore, we examined whether histone H3K27ac was necessary for the establishment of chromatin architecture at putative enhancers in the 4 hpf embryo. Zebrafish embryos were treated with either vehicle (DMSO) or SGC-CBP30 (SGC), a bromodomain inhibitor of histone acetyltransferase Ep300a (EP300 human ortholog) and Crebbp (CREBBP [also known as CBP] human ortholog) starting at the one-cell stage and continuing through ZGA. The 4 hpf treated embryos were collected for analysis by in situ Hi-C (Fig. 4A; Supplemental Fig. S3B; Supplemental Table S1). We verified inhibition of Crebbp/Ep300a activity by the approximately twofold bulk reduction of histone H3K27ac by quantitating loss of H3K27ac on a western blot analysis (Supplemental Fig. S6A). We further verified a strong (four- to sixfold) focal reduction of histone H3K27ac at several SE regions, as assessed by ChIP-qPCR (Supplemental Fig. S6B), compared to the vehicle control. Upon treatment with SGC, we observed a loss of chromatin interactions (reduction of insulation score) across the putative SE and Group 3 enhancers compared to the vehicle control, whereas there was little impact on Group 1 and Group 2 insulation (Fig. 4A). To examine the relationship between enhancers and adjacent boundaries, we assessed the strength of the nearest boundaries for each enhancer at 4 hpf upon SGC treatment; we observed little change in the negative insulation score compared to the vehicle control (Supplemental Fig. S6C). Additionally, by reanalyzing published Click-iT-seq data, upon SGC treatment the SE and Group 3 regions displayed a loss of transcription compared to control samples (Fig. 4B). Together these data suggest that Crebbp/Ep300a activity and subsequent histone H3K27ac are necessary for proper early embryo chromatin interactions at putative strong enhancers; however, diminishing H3K27ac does not affect the establishment of nearby boundaries (see Discussion, and Fig. 4C).

### Zebrafish sperm chromatin architecture has a unique configuration

**Figure 5.**
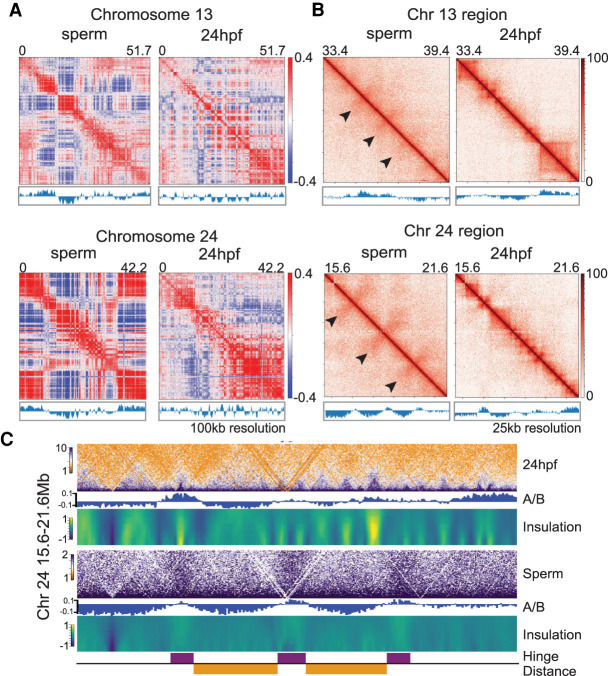
Hi-C contact maps in zebrafish sperm display “hinge-like” domains. (*A*) Correlation matrix from Chr 13 (*top*) and Chr 24 (*bottom*) for zebrafish sperm (*left*) and 24 hpf (*right*). First principal component values to determine A/B compartment status (*below*). (*B*) Contact matrices from two regions Chr 13: 33.4–39.4 Mb (*top*) and Chr 24: 15.6–21.6 Mb (*bottom*) for sperm (*left*) and 24 hpf (*right*), 25-kb resolution in log scale. Flares detected in sperm are marked by black arrows and are not evident in 24 hpf embryos. (*C*) Contact maps for 24 hpf and sperm samples each presented for a 6-Mb region on Chr 24 at 25-kb resolution in log scale (*top*). First principal component values to determine A/B compartment status (*middle*). Heatmap of insulation scores for different window sizes (*bottom*). The hinge region is marked by a purple square, and distance is marked by an orange square.

**Figure 6.**
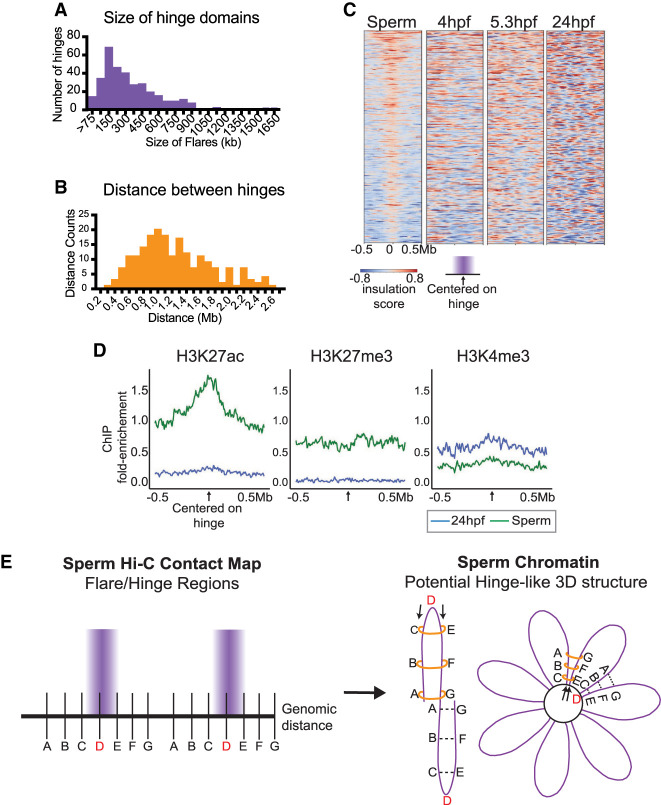
Global characterization of “hinge-like” domains in sperm chromatin architecture. (*A*) Histogram to depict the width distribution of the 333 hinges. (*B*) Histogram to depict the average distances between adjacent hinges. (*C*) Insulation scores at hinge-like domains in sperm, and insulation scores at the corresponding regions in embryos at 4, 5.3, and 24 hpf. Positive insulation (red) indicates increased contacts, and negative insulation (blue) indicates a lack of contacts. (*D*) Metaplot of log_2_ fold enrichment of histone H3K27ac (Zhang et al. 2018), H3K27me3 (Irimia et al. 2012), and H3K4me3 (Zhang et al. 2016) ChIP-seq signal over input for sperm (green) and 24 hpf (blue) centered on the hinge-like domains. (*E*) Two schematics depict two speculative models of zebrafish sperm chromatin and hinge architecture. In both models, the sperm DNA (which is nucleosome-packaged, not protamine-packaged) is arranged into arrays of consecutive loops/petals, possibly similar to the loops described for condensed mitotic chromosomes in somatic cells (Gibcus et al. 2018). Within each petal DNA positions A–G represent the repeated “hinge” unit within each petal, with “D” representing the hinge center, and the segments A–C and E–G representing the edges of the hinge “petals.” A key feature of the contact map data is that locations equidistant from position D show increased interaction. Two models are presented to achieve this: (1) interactions are caused by contacts *within* each petal (*left*), or (2) interactions are caused by contacts *between* two petals (*right*). In both models, we propose a constraint on topology, which might involve the loading of ring-like proteins (e.g., condensin or cohesin complexes) at the hinge position D (*left*) which, together with the fixed hinge position D, create the hinge-like domain through loop extrusion. Ring-like proteins are represented by orange rings. Arrows on the *left* indicate potential locations where ring-like proteins might load, at hinge position D, to help form stable hinges.

Lastly, we explored the unique structures observed in the zebrafish sperm Hi-C contact maps. We compared our sperm and 24 hpf contact maps, because prior work in mice and rhesus monkey reported strong similarities between somatic cells and sperm cells (Battulin et al. 2015; Jung et al. 2017; Wang et al. 2019). First, genome A/B compartment calls (and their boundaries) between sperm and 24 hpf were largely nonoverlapping (Fig. 5A). The sperm contacts display a peak distance of interaction >1 Mb (Supplemental Fig. S7A), which in somatic cells has been interpreted as signifying chromosome condensation (Naumova et al. 2013; Hug et al. 2017; Gibcus et al. 2018). Furthermore, whereas the 24 hpf Hi-C data contact maps showed traditional TADs with enhanced contact frequency, sperm lacked TAD structures (Fig. 5B). Instead, sperm displayed a unique feature that resembles a “flare”; a feature that is perpendicular to the diagonal in the contact maps (Fig. 5B,C; for additional examples, see Supplemental Fig. S7B). The flare feature is consistent with a large region displaying increased interactions primarily between locations equidistant from a fixed pivot/hinge point. This raises the possibility of periodic self-associating “hinge-like” chromosome domains occurring throughout the sperm genome (Fig. 6E).

To quantify the attributes of flares, we devised a computational strategy using the insulation score (Methods) to extract them, yielding a total of 333 flares across the zebrafish sperm genome. These flares range in size, although in aggregate generate a unimodal peak centered at ∼150 kb (Fig. 6A). After filtering out genome scaffolding errors, the distance between flare domains revealed a periodicity of approximately 1 Mb, indicating chromosome structure at the megabase scale (Fig. 6B). These flare domains are unique to sperm because the insulation score formed across each flare location was not observed at 4, 5.3, or 24 hpf (Fig. 6C).

To further characterize flares, we determined whether particular chromatin features were correlated with flares—including histone modifications, cohesin complex, gene density, repeat regions, and evolutionary breakpoints. Regarding cohesin, the Rad21 subunit was not detectable by western analysis in sperm (but was clearly detected at 4 and 24 hpf), but the Smc3 subunit was detectable (Supplemental Fig. S7B), as expected owing to the variety of spermatogenesis-specific cohesin complexes (Hopkins et al. 2014; Biswas et al. 2016). However, we observed no focal enrichment of Smc3 occupancy on sperm chromosomes by ChIP-seq analysis (Supplemental Fig. S7C). Furthermore, we found no enrichment of repetitive elements at flares, nor an increase in GC percent distribution (Supplemental Fig. S8A,B). Evolutionary breakpoints have overlap with 24 hpf boundaries (Supplemental Fig. S8C; Yang et al. 2020) but not in sperm. Transcription start sites (TSS) are enriched in flares, but they are not significantly used for early embryo gene expression (ZGA TSS), suggesting that flares in sperm are not pre-marking early embryonic expression (Supplemental Fig. S8D,E). Last, we analyzed available genome-wide chromatin immunoprecipitation sequencing (ChIP-seq) data, derived from sperm and 24 hpf samples. Histone H3K27ac (Zhang et al. 2018) was often enriched across flare regions in sperm, whereas H3K27ac was not enriched at these regions at 24 hpf (Zhang et al. 2018). In contrast, histone H3K4me3 (Zhang et al. 2016) and H3K27me3 (Irimia et al. 2012) showed little enrichment at flares (Fig. 6D). In summary, only H3K27ac and TSS locations show positive correlation with flares.

Taken together, our analysis of mature sperm suggests that chromatin folding in sperm is neither random nor similar to somatic patterns; it may instead involve the partitioning of the genome into “hinge-like” domains. One model consistent with our data involves these “hinge-like” regions arranging in a manner similar to the mitotic flower spiral structure that has been proposed for mitotic chromosomes (Naumova et al. 2013; Gibcus et al. 2018). In adapting this model to sperm chromatin, the “hinge-like” regions might form arrays of consecutive loops/petals, where point “D” represents the center of the hinge, and segments A–C and E–G represent the edges or “hinge” petals (Fig. 6E). In this model, the flare appears on the Hi-C contact map because locations equidistant from position D are more often in physical proximity.

## Discussion

The process of ZGA involves the proper activation of many housekeeping genes and the proper regulation (activation or silencing) of many developmental genes. The initial transcription at ZGA could (hypothetically) benefit from the utilization of chromatin architectural elements such as A/B compartments, TADs, and enhancer–promoter loops, and these elements in the embryo could (in principle) derive from states inherited from the parental gametes. However, zebrafish ZGA occurs during cleavage stage, which is characterized by rapid replication and about 10 synchronous cell division cycles that average ∼15 min. The major wave of ZGA occurs at ∼3–4 hpf, as cell cycles begin to lengthen and lose their synchrony (Lee et al. 2013). Thus, the process of ZGA co-occurs with rapid replication and cell division, processes which might impose physical and/or kinetic barriers to the establishment of particular chromatin architectural elements. In this study, we explored the similarities and major differences with prior studies of higher-order chromatin architecture in the zebrafish embryo, provided new information on enhancer interactions, and revealed unique architectural features in the zebrafish sperm that advance our understanding of the logic and use of chromosome architecture in zebrafish gametes and embryos.

First, we do not observe higher-order structure elements, such as A/B compartments or TADs, in pre-ZGA and ZGA embryos (Fig. 1B,C). Our work here, coupled to collaborative work (Nakamura et al. 2021), suggests that the prior observation of structure may have been caused by contamination. We hypothesize that the somatic cell contamination involves maternal oocyte follicle cells that surround the chorion during oocyte maturation (although other sources of contamination are not ruled out, such as adult fin tissue). We speculate that the use of a late dechorionation step leads to these somatic cells being released from the outer chorion surface and subsequent association with the embryo. The reported structure during pre-ZGA was diminished by ZGA, which may be explained by dilution of maternally derived cells compared to embryo-derived cells during subsequent rapid embryonic cell cycles. We also ruled out an alternative hypothesis that the observed structure pre-ZGA derived from contaminating sperm cells by demonstrating that sperm cells lack the observed pre-ZGA structures (Figs. 1B, 2D).

Our work instead supports a model through which the process of rapid replication and cell division in the early zebrafish embryo may be incompatible with the formation and utilization of higher-order chromatin structure; therefore, higher-order structural elements only emerge gradually, after ZGA. Additionally, the lack of structure before ZGA has been observed previously in *Drosophila*, another species with fast cycling cells during early embryogenesis (Hug et al. 2017). In mammals, chromatin architecture in early embryos is limited to focal regions and is not fully established genome-wide until after ZGA (Du et al. 2017; Hug et al. 2017). These results in embryos are consistent with observations in cultured mammalian cells, which showed that the reestablishment of higher-order chromatin structures after mitosis occurs on the “hours” timescale (Abramo et al. 2019; Zhang et al. 2019). However, a subset of boundaries, as well as the formation of smaller domains (involving enhancers with high H3K27ac), form relatively quickly after mitosis (Zhang et al. 2019). Following cohesin loss, although TADs largely disappear, regions with high histone H3K27ac such as super-enhancers, retain and even increase their associations, which may involve both inter- and intra-enhancer interactions (Rao et al. 2017; Rosencrance et al. 2020). However, in normal cycling cells, these sub-TAD size interactions have been shown to have Med12/cohesin (lacking CTCF) (Phillips-Cremins et al. 2013). These observations in cultured mammalian cells may relate to our observations of initial small interacting domains forming in the cycling zebrafish embryo at regions bearing high H3K27ac and cohesin, most prominently at candidate zebrafish super-enhancers (Fig. 3B). We propose that higher-order, 3D structure (beyond small contact domains) does not form in the zebrafish embryo until after ZGA, likely owing to the restrictions imposed by the fast cell cycle of the pre-ZGA embryo. Additionally, cohesin/Rad21 is absent from genes pre-ZGA and is redistributed from heterochromatic regions to genes post-ZGA. Further supporting our observation of a lack of structure pre-ZGA (Meier et al. 2018). Following, ZGA, chromatin architecture begins at enhancer regions bearing high H3K27ac, largely independent of transcription, including candidate super-enhancers—possibly to facilitate the subsequent formation of enhancer–promoter loops during the upcoming stages of development (Fig. 3B). Finally, we note that prior observations of TAD structure in mammalian cleavage-stage embryos is not in conflict with our observations in zebrafish, because mammalian embryos have much longer cell cycles during cleavage stage (12–24 h) than do zebrafish (∼15 min) (Ke et al. 2017; Collombet et al. 2020). This reflects the very different fates of (and threats to) these two types of embryos: mammalian embryos slowly progress to implantation, whereas fish embryos must rapidly progress to free-swimming fish to avoid predation.

We provide the first Hi-C analysis of sperm in a vertebrate that packages its genome entirely in histone rather than primarily small basic proteins such as protamine. Indeed, zebrafish lack a gene encoding protamine or protamine-like proteins and instead use typical somatic histones along with a high level of H1-family linker histone (as well as the H2A variants H2A.Z/FV and H2A.FX) as the most prominent proteins for packaging the genome in a condensed manner (Wu et al. 2011; Murphy et al. 2018). Our work suggests a lack of TAD structures (Figs. 1B, 5B,C; Supplemental Fig. S1C,D), but the presence of apparent A/B compartments in sperm (Figs. 1C, 5A). We emphasize that mature sperm have ceased transcription, and therefore the traditional notion that A/B compartments define an active or inactive genome does not apply to the sperm compartments but instead reflects the underlying principal component analysis (PCA) used to define them. The PCA method strictly segregates two types of genomic regions that alternate along the length of chromosomes, which in sperm is largely defined as regions within or outside of a “flare.” Flares are the sole visual feature that we observe in the contact maps from sperm (Figs. 1B, 5B), which appear perpendicular to the diagonal in the contact maps. Notably, we found enrichment of H3K27ac in flares, however functional experiments will be required to test whether H3K27ac is necessary for flare formation. We suggest that these flares represent physical “hinge-like” domains. Flares/hinges occur when two regions equidistant from a “fixed point” on the chromosome are much more likely to be in contact with each other than with any other region between them, including the fixed point. This fixed point is located at the center of the flare and might function physically as a hinge, with the flanking chromosomal regions folded over one another. The fixed point D represents the center of the hinge, and segments A–C and E–G represent the edges or petals (Fig. 6E). We hypothesize that these hinge-like domains are formed to facilitate the compaction of the histone-bound DNA into the sperm head.

Additional topological constraints are needed to favor equidistant interactions, and we propose two speculative models to achieve this: an “intra-loop model” favors interactions between regions equidistant from point D, although solely within the loop (Fig. 6E, left), whereas an “inter-loop model” also favors interactions between regions equidistant from point D, but involves adjacent loops (Fig. 6E, right), possibly arranged on a central scaffold. Furthermore, both models can accommodate a role for two-sided loop extrusion using cohesin to constrain the structured loop (Fudenberg et al. 2017; Banigan et al. 2020). Two-sided loop extrusion would create and stabilize the hinge-like domain with equidistant interactions, which may be established or maintained through the loading of cohesin from the hinge fixed point D (Banigan et al. 2020). We note that the different topological constraints needed to form hinge-like domains could (in principle) be facilitated by any one of the five different cohesin complexes, formed by the exchange of the individual subunits within the cohesin complex, during spermatogenesis (Hopkins et al. 2014; Biswas et al. 2016). Zebrafish sperm chromatin architecture could also use mitotic-/meiotic-specific proteins, such as condensins (Gibcus et al. 2018), to create the “hinge-like” regions. Any of these structural proteins could create a mitotic flower spiral structure similar to that proposed for the condensed mitotic chromosome (Naumova et al. 2013; Gibcus et al. 2018). Additionally, we speculate that the inability to detect flares in traditional Hi-C contact maps of mitotic cells may result from an averaging of signal between two sister chromatids, and potentially that mitotic cells may have a higher degree of condensation than does zebrafish sperm. Future work examining the possible roles of candidate factors in flare formation in sperm will help clarify the structural basis for flares in sperm and test the “hinge model” of genome packaging for the histone-packaged zebrafish sperm genome.

## Methods

### Experimental models and subject details

#### Zebrafish embryo culture

Zebrafish *Danio rerio* strains were maintained under accordance with approved institutional protocols at the University of Utah (Westerfield 2000). All experiments using zebrafish were approved by IACUC Protocol 17-01006 and 20-04011. Wild-type zebrafish were from the Tübingen (Tü) strain, and Wik strain. Experimental samples were either mature gametes (sperm) or early zebrafish embryos ranged from 0 to 24 hpf. Live embryos were maintained at 28.5°C. All developmental staging was based on hours after post-fertilization and visual confirmation of timing.

#### Drosophila S2 cells

Schneider S2 cells derived from *D. melanogaster* were cultured in Gibco Schneider's *Drosophila* medium (Thermo Fisher Scientific 21720024) supplemented with 10% FBS (Omega Scientific FB-11) and penicillin/streptomycin. Cells were grown at room temperature and split as the dish became confluent.

### Method details

#### Isolating zebrafish cells from embryo and sperm for Hi-C protocol

##### Early dechorionated pre-ZGA samples

Pre-ZGA samples were dechorionated with pronase (Sigma-Aldrich, working concentration 10 mg/mL) at one-cell stage shortly after mating. For full details, see Supplemental Methods.

##### Late dechorionated pre-ZGA, 4, 5.3, and 24 hpf samples

Embryos at pre-ZGA, 4, 5.3, and 24 hpf were dechorionated with pronase at the time of collection as described above. After the 4–5 washes, the embryos were transferred carefully to a 1.5 mL eppendorf tube with a transfer pipette to not disrupt the embryos. The embryos were then deyolked because the yolk proteins interfere with digestion steps later in the Hi-C protocol. See Supplemental Methods for more details.

##### Collection and fixation of sperm samples

Sperm samples were collected with standard methods as previously described (Kroeger et al. 2014) and fixed in 1% formaldehyde for 10 min at room temperature and stopped with 0.2 M glycine. Sperm cells were washed in 1× PBS and snap frozen with liquid nitrogen and stored at −80°C.

#### Fixing S2 cells for Hi-C protocol

For spike-in preparation, standard fixation methods were used. See Supplemental Methods for more details.

#### Embryo inhibitor treatment

Flavopiridol (Selleck Chemicals, final 1.5 µM) and SGC-CBP30 (Sigma-Aldrich, final 20 µg/µL) were prepared in DMSO. Embryos were incubated at indicated concentrations in E3 embryo water (Westerfield 2000) for 4 h at 28.5°C. Controls were incubated in (1%) DMSO, in E3 (vehicle).

#### Hi-C protocol

##### Isolating zebrafish embryo nuclei

Aliquots of enough cells at each time point were pulled out of the freezer; 2.25 hpf (10,000 to 100,000 cells), 4 hpf (500,000 cells), 5.3 hpf (400 embryos, ∼1 million cells), and 24 hpf (40 embryos, ∼1 million cells). Cells were thawed on ice and recounted to verify accurate spike-in amount. Zebrafish cells were washed one time with Hi-C lysis buffer (10 mM Tris-Cl, pH 8.0, 10 mM NaCl, 0.2% IGEPAL CA-630) followed by a 15-min lysis incubation on ice. During the lysis step, the *Drosophila* S2 cells were added to each sample to equal 1/5 of the zebrafish cell count.

##### Isolating zebrafish sperm nuclei

An aliquot of cells was thawed on ice and recounted to verify accurate spike-in amount. Approximately 4 million cells were used per sample. Cells were washed one time with Hi-C lysis buffer followed by a 15-min lysis incubation on ice. During the lysis step, the *Drosophila* S2 cells were added to each sample to equal one-fifth of the zebrafish cell count.

##### Isolating Drosophila S2 cells

Aliquots of cells were thawed on ice and recounted to verify accurate spike-in amount. No more than 5 million cells were lysed at one time using 500 µL Hi-C lysis buffer on ice. Once the S2 cells were resuspended in lysis buffer, they were added to the zebrafish cells undergoing lysis at the same time. See Supplemental Table S1 for all replicates where S2 cells were included.

##### Low-cell in situ Hi-C after cell lysis

Following nuclei isolation, the standard operating practices of the 4DN in situ Hi-C protocol was followed (Rao et al. 2014) adjusting buffers/enzymes based on the protocol for low-cell in situ Hi-C (Díaz et al. 2018). For details on the Hi-C protocol and library production, see Supplemental Methods.

#### ChIP-seq protocol

ChIP experiments were carried out as described previously (Goren et al. 2010), modified for cell isolation from the zebrafish sperm or embryos. For full details, see Supplemental Methods.

#### ATAC-seq protocol

The original protocol (Buenrostro et al. 2015) was modified for zebrafish nuclei collection. For full details, see Supplemental Methods.

#### Immunohistochemistry and DAPI staining early zebrafish embryos

Standard protocol for immunohistochemistry was followed (Zhang et al. 2018). For full details, see Supplemental Methods.

#### Imaging zebrafish embryos

Confocal images were acquired on a Leica SP8 White Light laser confocal. Image processing was completed using Nikon NIS-Elements multiplatform acquisition software with a 40×/1.10 Water objective. Fiji (ImageJ, V 2.0.0-rc-69/1.52p) was used to color DAPI channel to cyan, GFP color remained green. Confocal images are max projections of Z stacks taken 0.5 μm apart for a total of the embryo ∼7–12 μm. See Supplemental Methods for the description for DAPI staining and cell cycle staging for the 2.25 hpf Hi-C embryo samples.

### Quantifications and statistical analyses

#### Hi-C data processing

Reads were aligned to a merged Zv10 (chromosomes were labeled 1–25) and dm6 (chromosomes were labeled 2L, 2R, 3L, 3R, 4D, XD, YD) genome using BWA-MEM (V 0.7.15-r1140, http://bio-bwa.sourceforge.net/bwa.shtml) using the following options -A 1 -B 4 -E 50 -L 0. HiCExplorer (V3.3, https://hicexplorer.readthedocs.io/en/latest/) hicBuildMatrix was used to create matrix at 10, 25, and 50 kb resolutions, using the option –outBam (to extract valid Hi-C reads). For full details of Hi-C data processing, see Supplemental Methods.

#### Flare/hinge calling

Flare/hinge regions in sperm Hi-C data were found by extracting the positive values from the last column of the bedGraph matrix in the “tad_score.bm” file from hicFindTADs command HiCExplorer (V3.3). Flares/hinges were merged if within 50 kb of each other, and the first round of filtering was done to remove blacklisted regions as described previously. A second round of filtering was done by visually inspecting the positive flares and verifying they were not a false positive attributable to a genome assembly issue. Once flare/hinge list was filtered, the size of a flare was calculated by the width of the positive values in the bedGraph matrix. The distance between two flares was calculated by measuring the distance of one flare to the other. The distance between flares was excluded if there was a genome assembly gap creating a blacklisted region causing an inaccurate distance measurement.

#### ChIP-seq and ATAC-seq data processing and peak calling

FASTQ files were aligned to Zv10 using NovoAlign, and BAM files for technical replicates were merged using SAMtools merge (V1.8, http://www.htslib.org/doc/samtools-merge.html) (Li et al. 2009). Data with multiple biological replicates were then processed using Multi-Replica Macs ChIP-seq Wrapper. For full details, see Supplemental Methods.

#### ROSE enhancer algorithm and sequence motif enrichment analysis

To identify enhancers and super-enhancers (SEs), the ROSE algorithm version 0.1 was applied with default parameters performing TSS exclusion (–t 2000) (Lovén et al. 2013; Whyte et al. 2013). Using the intersected peaks between H3K4me1 and H3K27ac ChIP-seq signal (this list also excluded promoters). The 4 hpf enhancers were stratified into three equal-sized cohorts for further examination, and the super-enhancers remained at *N* = 411. For the 24 hpf potential enhancers, the enhancers list in Pérez-Rico et al. (2017) were lifted over using UCSC to Zv10 and used in the ROSE algorithm version 0.1. Candidate transcription factor motifs was determined by intersecting potential enhancers with ATAC-seq narrowpeaks signal, and this list was used in HOMER findMotifsGenome.pl to find known binding motifs and motif frequency. The list was cross-referenced with Click-iT-seq data, regardless of maternal contribution, to verify expression of potential transcription factor in the early embryo. Bubble plot was created in R (R Core Team 2017), using standard methods. Ctcf motifs were determined by HOMER findMotifsGenome.pl, potential Ctcf locations were determined by converting the HOMER output to WIG files and ran in Danpos (Chen et al. 2013) for locations.

#### Metaregion analysis for ChIP-seq, ATAC-seq, and Click-iT-seq

To generate metaregions plots of ChIP-seq, ATAC-seq, and Click-iT-seq, signal was averaged in 10-kb bins across the genome using get_datasets.pl from Biotoolbox (https://metacpan.org/release/Bio-ToolBox). The metaplots were visualized in R using standard methods. For full details, see Supplemental Methods.

## Data access

All raw and processed sequencing data generated in this study have been submitted to the NCBI Gene Expression Omnibus (GEO; https://www.ncbi.nlm.nih.gov/geo/) under accession number GSE152744. The Hi-C contact maps and raw data in this study have also been submitted to the 4D Nucleome data portal (https://data.4dnucleome.org/Cairns_zf_embryo_HiC).

## Supplementary Material

Supplemental Material
