# Supplementary material for: Chromatin architecture transitions from zebrafish sperm through early embryogenesis
Source: Genome Res. 2021 Jun;31(6):981–94. doi: 10.1101/gr.269860.120 (PMC8168589; doi:10.1101/gr.269860.120)
Supplement: Supplemental Material [file supp_31_6_981__DC1.html]

Chromatin architecture transitions from zebrafish sperm through early embryogenesis — Chromatin architecture transitions from zebrafish sperm through early embryogenesis — Supplemental Material 

# Chromatin architecture transitions from zebrafish sperm through early embryogenesis

## Supplemental Material

- Supplemental\_Table\_S1.xlsx
- Supplemental\_Material\_.docx
