# Supplementary material for: Chromatin architecture transitions from zebrafish sperm through early embryogenesis
Source: Genome Res. 2021 Jun;31(6):981–94. doi: 10.1101/gr.269860.120 (PMC8168589; doi:10.1101/gr.269860.120)
Supplement: Supplemental Material [file supp_gr.269860.120_Supplemental_Material_.docx]

**Supplemental material**

**Chromatin architecture transitions from zebrafish sperm through early embryogenesis**

Candice Wike^1^, Yixuan Guo^1^, Mengyao Tan^1^, Ryohei Nakamura^2^, Dana Klatt Shaw^3^, Noelia Díaz^4^, Aneasha F. Whittaker-Tademy^1^, Neva C. Durand^5^, Erez Lieberman Aiden^5^, Juan Vaquerizas^4,6^, David Grunwald^3^, Hiroyuki Takeda^2^, & Bradley R. Cairns^1*^

^1^Department of Oncological Sciences, Huntsman Cancer Institute and Howard Hughes Medical Institute, Salt Lake City, UT 84112, USA.

^2^Department of Biological Sciences, Graduate School of Science, The University of Tokyo, Tokyo 113-0033 Japan.

^3^ Department of Human Genetics, University of Utah, Salt Lake City, UT 84112, USA.

^4^Max Planck Institute for Molecular Biomedicine, Roentgenstrasse 20, 48149 Muenster, Germany

^5^The Center for Genome Architecture, Baylor College of Medicine, Houston, TX 77030, USA

Department of Molecular and Human Genetics, Baylor College of Medicine, Houston, TX 77030, USA. Department of Computer Science, Department of Computational and Applied Mathematics, Rice University, Houston, TX 77005, USA. Broad Institute of Harvard and Massachusetts Institute of Technology (MIT), Cambridge, MA 02139, USA. Center for Theoretical Biological Physics, Rice University, Houston, TX 77030, USA.

^6^MRC London Institute of Medical Sciences, Du Cane Road, London W12 0NN, UK. Institute of Clinical Sciences, Faculty of Medicine, Imperial College London, Hammersmith Campus, Du Cane Road, London W12 0NN, UK.

*Correspondence to; Bradley R. Cairns (brad.cairns@hci.utah.edu)

Table of contents

Pages 2-18: Supplemental Methods

Pages 19-32: Supplemental Figures & legends

Pages 33-34: Supplemental References

Additional File: Supplemental_Table_S1.xlsx

**SUPPLEMENTAL METHODS**

**Isolating zebrafish cells from embryo and sperm for Hi-C protocol**

*Early dechorionated preZGA samples*

PreZGA samples were dechorionated with pronase (Sigma-Aldrich, working concentration 10mg/ml) at 1-cell stage shortly after mating. About 500 µL of pronase was dropped onto embryos in 10cm dish with just enough E3 buffer to cover the embryos for 7 mins. After the 7 mins the pronase+E3 buffer was poured off of the embryos carefully and more E3 buffer (pre-warmed to 28.5°C) was added to the dish for a wash. Embryos were kept under buffer the entirety of the washing to prevent any bursting. The embryos were swirled with the E3 buffer a few times and the buffer was then poured off. The wash procedure was repeated 2-3 times until chorions were no longer visible in the buffer or surrounding embryos. The embryos were then transferred to a glass dish and washed an additional two times with more E3 buffer. Embryos were then incubated at 28.5°C until collection time. At the time of collection, the embryos were transferred to 1.5 mL eppendorf tube with a transfer pipette carefully to not disrupt the embryos.

*Late dechorionated preZGA, 4hpf, 5.3hpf, and 24hpf samples*

Embryos at preZGA, 4hpf, 5.3hpf and 24hpf were dechorionated with pronase at the time of collection as described above. After the 4-5 washes the embryos were transferred to 1.5 mL eppendorf tube with a transfer pipette carefully to not disrupt the embryos. The embryos were then deyolked as the yolk proteins interfere with digestion steps later in the Hi-C protocol.

The 4hpf, 5.3hpf and 24hpf samples were deyolked with the deyolking buffer (55mM NaCl, 1.8mM KCl, 1.25mM sodium bicarbonate) and both early and late dechorionated preZGA samples were deyolked with 0.5M sucrose/PBS to preserve cell membrane integrity. Zebrafish embryo cells were fixed using 1% formaldehyde and 1xPBS for 10mins at room temperature and stopped with 0.2M glycine. Cells were washed in 1x PBS and snap frozen with liquid nitrogen and stored at -80°C.

*Collection and fixation of sperm samples*

Sperm samples were collected and put into Hank's balanced salt solution (Kroeger et al. 2014) kept on ice and counted with hemocytometer. Approximately 5 million sperm cells per tube were aliquoted, then washed twice in 1xPBS and fixed in 1% formaldehyde for 10mins at room temperature and stopped with 0.2M glycine. Sperm cells were washed in 1x PBS and snap frozen with liquid nitrogen and stored at -80°C.

**Fixing S2 cells for Hi-C protocol**

For spike-in preparation, S2 cells were counted with a hemocytometer and split into separate tubes with a maximum of 5million cells per tube, washed twice in 1xPBS and fixed with 1% formaldehyde/1xPBS for 10mins. Fixation was stopped with 0.2M glycine for 5 mins. The aliquots of cells were then pelleted, flash frozen in liquid nitrogen, and stored in -80°C, until utilized in Hi-C experiments. Important to note, adding the spike-in control at the cell lysis, doesn’t account for differences such as fixation, however, we try and alleviate this by fixing S2 cells in batches of no more than 5 million cells.

**Hi-C protocol**

*Isolating zebrafish embryo nuclei*

Aliquots of enough cells at each timepoint were pulled out of freezer; 2.25hpf (10K to 100K cells), 4hpf (500K cells), 5.3hpf (400embryos, ~1 million cells) 24hpf (40embryos, ~1 million cells), Sperm (~ 1million cells). Cells were thawed on ice and recounted to verify accurate spike-in amount. Zebrafish cells were washed one time with Hi-C lysis buffer (10 mM Tris-Cl pH 8.0, 10 mM NaCl, 0.2% IGEPAL CA-630) followed by a 15min lysis incubation on ice. During the lysis step the *Drosophila* S2 cells were added to each sample to equal 1/5 of the zebrafish cell count.

*Isolating zebrafish sperm nuclei*

An aliquot of cells was thawed on ice and recounted to verify accurate spike-in amount. Approximately 4 million cells were used per sample. Cells were washed one time with Hi-C lysis buffer followed by a 15min lysis incubation on ice. During the lysis step the *Drosophila* S2 cells were added to each sample to equal 1/5 of the zebrafish cell count.

*Isolating Drosophila S2 cells*

Aliquots of cells were thawed on ice and recounted to verify accurate spike-in amount. No more than 5 million cells were lysed at one time utilizing 500µl Hi-C lysis buffer on ice. Once the S2 cells were resuspended in lysis buffer they were added to the zebrafish cells undergoing lysis at the same time. Please see supplemental table 1 for all replicates where S2 Cells were included.

*Low cell in situ Hi-C after cell lysis*

Following nuclei isolation, the standard operating practices of the 4DN in situ Hi-C protocol was followed (Rao et al. 2014) adjusting buffers/enzymes based on the protocol for low cell in situ Hi-C **(Diaz et al. 2018).** Briefly, nuclei were washed in ice-cold 1X NEB buffer 2, followed by resuspension in 0.4% SDS incubated for 10mins at 65°C without agitation. SDS was quenched by adding 10% Triton X-100 and water, sample was then incubated at 37°C for 45mins at 650 rpm in an Eppendorf ThermoMixer. Prior to digestion, 10% of the volume was removed for quality control (QC) and digestion efficiency assays. MboI digestion was carried out in 1xNEB2 buffer for 90mins at 37°C with a gentle rotation. For 10K-100K cells 50units was used, for 100K-5M cells 100units was used. After digestion, 10% was collected for QC and digestion efficiency. Overhangs generated by restriction enzymes were filled-in using 1.0mM biotin-14-dATP (Axxora, JBS-NU-835-BIO14-L) and other appropriate nucleotides with DNA polymerase 1 Klenow Fragment enzyme (NEB, M0210L) for 90mins at 37°C. Ligation was completed using T4 DNA ligase (NEB, MO202L) and appropriate buffers. Samples were rotated for 4h at room temperature. Once complete 10% of supernatant and 10% of pellet were collected for QC. Finally, DNA was extracted using an extraction buffer (adding 30 μl of 10% SDS, 24 μl 5M NaCl to 500 μl TE buffer) per sample before use. proteinase K was added and samples were digested for 30mins at 55°C at 1000 rpm. Then incubated at 65°C at 1000 rpm overnight. Lastly, DNA was extracted with phenol‐chloroform, ethanol precipitated, and treated with RNase. Quality control samples collected throughout the Hi-C protocol was run on a 5x TBE gel to verify digestion and ligation, while maintaining nuclei integrity. Digestion efficiency was detected after the experiment was completed. It was performed as suggested in (Hagege et al. 2007) a sample was only proceeded to library prep if it had an 80% efficiency digestion rate.

*Library construction*

For library construction using the Illumina Hiseq 2500 instrument no more than 5 μg of DNA was used for the following steps. Biotin was removed from unligated fragments using T4 DNA Polymerase incubated for 4hrs at 20°C. Nuclei was pelleted and then resuspended in 130uL of Nuclear Lysis buffer (50 mM Tris-HCl pH 7.5, 10 mM EDTA, 1% SDS, 1x protease inhibitor). DNA was sheared on a S220 Focused-ultrasonicator (Covaris) using a microTUBE AFA Fiber Pre-Slit Snap-Cap (Covaris, Cat# 520045) with the following program (4 cycles, each 60 secs long, 5% duty, 4 intensity, 200cycles/burst). The ligated biotin junctions were pulled down utilizing Dynabeads My one Streptavidin C1 (Life technologies, Cat# 65001). Library construction was then performed on beads by resuspending 1X TD (10 mM Tris-HCl, pH=8.0, 5 mM MgCl2; 10% DMF) add no more than 2.5 μl home-made Tn5 assembly (Picelli et al. 2014). The Tn5 assembly amount used was dependened on the batch’s activity at the time of use, which was optimized in a separate mock Hi-C experiment. Samples were incubated at 55°C for 10mins. The Tn5 library construction reaction was stopped with 0.5 μl 10% SDS on ice for 5 mins. Samples were than washed two times in 50mM EDTA followed by one wash in 10 mM Tris-HCl (pH 8.0). The library was resuspended in 22μl EB buffer. The beads can inhibit the PCR steps so the resuspended beads were split into 2 reactions with 10 μl of resuspended beads per reaction. Library construction PCR was done utilizing 2x Phusion High Fidelity PCR master Mix with HF Buffer (NEB cat #M0531S) and custom PCR primers (supplemental table 2). To determine the appropriate number of cycles a “pre-amp” PCR reaction was performed where 5 cycles of the PCR reaction was performed and then 5 μl of the PCR reaction was used in a qPCR (utilizing iTaq Universal SYBR Green Supermix (Bio-Rad) using a BioRad qPCR CFX connect instrument) to determine the number of cycles before max absorbency was reached. Typically, no more than 7-13 additional cycles were performed for the library construction PCR reaction. The PCR libraries were then size selected with AMPure beads and quantified on an Agilent Technologies 2200 TapeStation with a D1000 ScreenTape assay. The resulting libraries were then sequenced on a 2 × 125 cycle paired-end run on an Illumina HiSeq 2500 instrument.

For library construction using the Illumina Nova-seq instrument no more than 5 μg of DNA was used for the following steps. Please note, the library construction protocol was changed when we switched to the Novaseq instrument due to the limitations of the machine we had to adapt and utilize a dual index primer set. Biotin was removed from unligated fragments using T4 DNA Polymerase incubated for 4hrs at 20°C. Nuclei was pelleted and then resuspended in 130uL of Nuclear Lysis buffer (50 mM Tris-HCl pH 7.5, 10 mM EDTA, 1% SDS, 1x protease inhibitor). DNA was sheared on a S220 Focused-ultrasonicator (Covaris) using a microTUBE AFA Fiber Pre-Slit Snap-Cap (Covaris, Cat# 520045) with the following program (2 cycles, each 50 sec long (total 100 secs), 10% duty, 4 intensity, 200cycles/burst). The ligated biotin junctions were pulled down utilizing Dynabeads My one Streptavidin C1 (Life technologies, Cat# 65001). Library construction was then performed on beads as instructed by NEBNext ChIP-seq Library Prep Master Mix Set for Illumina (E6240) with NEBNext® Multiplex Oligos for Illumina (Dual Index Primers Set, E7600S). Instead of AMPure bead washes between each library step the resuspended beads were washed using a magnet with the following steps: wash with 200 μl 1xBW (5 mM Tris-HCl (pH 7.5), 0.5 mM EDTA,1 M NaCl) and 0.1% Triton x-100. Second wash with 200 μl 10mM Tris-HCl (pH 7.5). When washes were finished the beads were resuspended in appropriate amounts of 10mM Tris-HCl (pH 7.5) required for the next step in the protocol. After the adaptor was ligated samples were resuspended in 50 μl 10mM Tris-HCl (pH 8.0) and proceeded to PCR library prep. The beads can inhibit the PCR steps so the resuspended beads were split into 5 reactions with 10 μl of resuspended beads per reaction. Library construction PCR was done utilizing 2x Phusion High Fidelity PCR master Mix with HF Buffer (NEB cat #M0531S) and NEBNext® Multiplex Oligos for Illumina (Dual Index Primers Set, E7600S). To determine the appropriate number of cycles a “pre-amp” PCR reaction was performed where 5 cycles of the PCR reaction was performed and then 5 μl of the PCR reaction was used in a qPCR (utilizing iTaq Universal SYBR Green Supermix (Bio-Rad) using a BioRad qPCR CFX connect instrument) to determine the number of cycles before max absorbency was reached. Typically, no more than 7-13 additional cycles were performed for the library construction PCR reaction. The PCR libraries were then size selected with AMPure beads and quantified on an Agilent Technologies 2200 TapeStation with a D1000 ScreenTape assay. The resulting libraries were then sequenced on a 2 × 150 cycle paired-end run on an Illumina Nova-seq instrument.

**ChIP-seq protocol**

ChIP experiments were carried out as described previously (Goren et al. 2010).

*Modified for cell isolation from the 4hpf and 24hpf embryos*. Briefly, approximately 200-300 embryos were dechorionated with pronase. Zebrafish cells were fixed using 1% formaldehyde and stopped with 0.2M glycine. Cells were snap frozen with liquid nitrogen and stored at -80°C. Approximately 1000 embryos were used for the 4hpf, and 200 embryos were used for the 24hpf. ChIP-seq by first lysing cells on ice and then DNA was fragmented to a size range of 200–700bp with a Branson 450 Digital Sonifier.

*Modified for sperm chromatin shearing*

Traditional Branson sonication did not shear zebrafish sperm chromatin. Following standard HiC preparation we were able to shear the sperm chromatin to the appropriate size range for ChIP. Sperm samples were collected and fixed as described above. Sperm cells were washed one time with Hi-C lysis buffer (10 mM Tris-Cl pH 8.0, 10 mM NaCl, 0.2% IGEPAL CA-630) followed by a 15min lysis incubation on ice. Nuclei were washed in ice-cold 1X NEB buffer 2, followed by resuspension in 0.5% SDS incubated for 10mins at 65°C without agitation. SDS was quenched by adding 10% Triton X-100 and water, sample was then pelleted at 2500xg for 5min at 4°C. Nuclei was resuspending in 130uL of Nuclear Lysis buffer (50 mM Tris-HCl pH 7.5, 10 mM EDTA, 1% SDS, 1x protease inhibitor). DNA was sheared on a S220 Focused-ultrasonicator (Covaris) using a microTUBE AFA Fiber Pre-Slit Snap-Cap (Covaris, Cat# 520045) with the following program (2 cycles, each 60 secs long, 5% duty, 4 intensity, 200cycles/burst). Samples were then transferred to a new tube and the SDS concentration was lowered to 0.1% using ChIP Dilution Buffer (0.01% SDS, 1.1% Triton X-100, 1.2 mM EDTA, 16.7 mM Tris-HCl pH 7.5, 167 mM NaCl) The samples were clarified by centrifugation for 15mins at 16100xg at 4°C. The solubilized chromatin was

*Antibody immunoprecipitation and washes*

A small amount of sample was taken as input. Solubilized chromatin was precleared with Protein A-Dynabeads for 1 hr at 4°C. The beads were removed and the solubilized chromatin was immunoprecipitated with antibody against H3K27ac (Active Motif, Cat # 39133), Pol II (RNA Pol II (8WG16/Covance MMS-126R), Smc3 (Cell Signaling, 5696S) or Rad21 (Abcam, Cat#992). Protein A‐Dynabeads were used to pull‐down the antibody–chromatin complexes which were then washed and eluted. After cross‐link reversal (5 hours, 65ºC) and proteinase K digestion, both immunoprecipitated DNA and input DNA were extracted with phenol‐chloroform, ethanol precipitated, and treated with RNase. Libraries were made by NEBNext ChIP-seq Library Prep Master Mix Set for Illumina (E6240) with NEBNext Multiplex Oligos for Illumina (E7335L). High-throughput sequencing was performed by Illumina’s protocol for 50bp single-end runs on an Illumina HiSeq 2500. Two biological replicates and one input were generated for the 4hpf timepoint. ChIP-qPCR was performed with the was performed with iTaq Universal SYBR Green Supermix (Bio-Rad) using a BioRad qPCR CFX connect instrument.

**ATAC-seq protocol**

The original protocol from (Buenrostro et al. 2015) was modified for zebrafish nuclei collection. Approximately 300 embryos/tube of a 4hpf embryo was lysed with Tn5 Lysis buffer (10mM Tris-Cl, pH 7.4, 10mM NaCl, 3 mM MgCl_2_, 0.1% Igepal CA 630 + 1% protease inhibitor). Nuclei were isolated by removing chorion, yolk and cell membrane by physical disruption utilizing a 3ml syringe/20G needle while kept on ice. Nuclei were washed 2 times at room temperature for 10mins with nuclei wash buffer (50mM Tris-HCl pH 8.0, 100mM NaCl, 10mM EDTA + 1% protease inhibitor). The ATAC-seq library was made using Nextera DNA Flex Library Prep Kit (illumina, 20018704) according to kit specifications. Proper sizing of tagmentation was determined by DNA acrylamide gels utilizing Typhoon imager (GE Healthcare) for each lot of enzyme used.

High-throughput sequencing was performed by Illumina’s protocol for 150bp paired-end runs on an Illumina HiSeq 2500. Two biological replicates were generated for the 4hpf timepoint.

**Western Blot**

Approximately 200-300 embryos were collected at the timepoints following the chorion removal. Samples were lysed and 4x SDS buffer was added to the sample to obtain a 1x final concentration and samples were boiled at 100°C for 5mins. The samples were then resolved on a 15% SDS-PAGE and transferred to nitrocellulose according to standard procedures. For Licor detection blots were blocked using 1% SeaBlock (Thermo Fisher Scientific, Cat # 37527). The blots were probed with primary antibodies (as listed above) at 4°C overnight. For loading controls the blots were probed with Histone H3 (mAb, Active Motif, Cat # 39763). Blots were washed and incubated in IRdye secondary antibodies (LICOR) at room temperature for 1 hr. Blots were scanned using the Odyssey® CLx Imaging System and it was used to analyze and quantitate bands.

**Immunohistochemistry and DAPI staining early zebrafish embryos**

Standard protocol for immunohistochemistry was followed as in (Zhang et al. 2018). Briefly, embryos were collected at appropriate time points and fixed with fresh 4% paraformaldehyde (Electron Microscopy, 50980487) in 1xPBS at room temperature for 2 hours or overnight at 4ºC. Fixed embryos were dehydrated in methanol and stored at -20ºC. For immune-staining, embryos were rehydrated into PB3T (1xPBS with 0.3% TritonX-100, and then incubated in blocking agent (1% BSA, 0.3 M glycine in PB3T). Embryos were incubated with primary antibodies diluted in blocking agent overnight at 4°C. Primary antibodies were removed and embryos were washed extensively with PB3T. Embryos were next incubated with appropriate secondary antibodies in the dark followed by extensive washes in PB3T. Primary antibodies used was: RNA Pol II ser 5 pho at 1:500 (Active Motif, Cat# 39233). Secondary antibodies used were donkey α-rabbit IgG-488 at 1:500 (Life Technologies, Cat # A-21206). DAPI was used at 1:1000 as a nuclear counterstain. The yolk cells were removed from embryo and embryo was mounted on glass slide with ProLong Gold Antifade mounting media (Thermo Fisher Scientific, Cat# P-36931) and a 2.0mm square coverslip sealed with nail polish. Samples were stored at 4°C until imaged.

**Imaging Zebrafish Embryos**

Confocal images were acquired on a Leica SP8 White Light laser confocal. Image processing was completed using Nikon NIS-Elements multi-platform acquisition software with a 40X/1.10 Water objective. Fiji (ImageJ, V 2.0.0-rc-69/1.52p) was utilized to color DAPI channel to cyan, GFP color remained green. Confocal images are max projections of Z stacks taken 0.5μm apart for a total of the embryo ~7-12 μm.

**DAPI staining and cell cycle staging for the 2.25hpf Hi-C embryo samples**

At the time of collecting 2.25hpf embryo samples for Hi-C approximately 25 embryos where collected and carefully labeled to reference back to the appropriate fixed Hi-C sample. The early dechorionated embryos were fixed at the same time as the Hi-C sample with 4% PFA overnight at 4°C. The late dechorionated embryos were fixed, with chorion on, at the same time as the Hi-C sample with 4% PFA overnight at 4°C. The embryos were manually dechorionated and washed in PB3T (as described above) for 5 mins. They were incubated with DAPI (1:500, 1mg/mL stock) for 30mins. Cell cycle stage (non-mitotic, mitotic, anaphase) for embryos in each 2.25hpf sample collection was counted utilizing an epifluorescence Zeiss Axioplan microscope (Zeiss). Representative images for Supplemental Fig. 1B were confocal images taken on Leica SP8 White Light laser confocal as described above. Samples with greater than 70% in a non-mitotic cell cycle stage were used for the 2.25hpf in the Hi-C samples.

**Embryo and sperm brightfield imaging**

Carefully staged embryos were imaged on an stereo dissecting microscope with brightfield and a 10x objective and imaged with Apple Iphone 10 through the microscope eyepiece. Images were converted to greyscale and brightness/contrast were adjusted linearly where appropriate in adobe photoshop. Sperm sample was imaged on inverted epifluorescence microscope (Zeiss). Brightness and contrast were adjusted linearly where appropriate in adobe photoshop.

**QUANTIFICATIONS AND STATISTICAL ANALYSES**

**Hi-C data processing**

Reads were aligned to a merged (chromosomes were labeled 1-25) and dm6 (chromosomes were labeled 2L, 2R, 3L, 3R, 4D, XD, YD) genome using BWA-MEM (V 0.7.15-r1140, http://bio-bwa.sourceforge.net/bwa.shtml) using the following options -A 1 -B 4 -E 50 -L 0. HiCExplorer (V3.3, https://hicexplorer.readthedocs.io/en/latest/) hicBuildMatrix was used to create matrix at 10kb, 25kb, 100kb resolutions, using the option –outBam (To extract valid Hi-C reads). Matrices of different species were split using hicAdjustMatrix. The replicates for each timepoint as demonstrated in supplemental table 1 were summed utilizing hicSumMatrices. Once summed the sperm, 2.25hpf, 4hpf, 5.3hpf, 24hpf matrices were normalized using hicNormalize option—smallest. The DMSO, SGC, and FLAV matrices were normalized to each other using hicNormalize --smallest. Filtering out low-count bins, the normalized matrices were corrected using hicCorrectMatrix. After correcting these final matrices were used for plotting images as well as for TAD/domain calling. For the processed files available on GEO, the valid pairs bam file from the 25kb built matrices replicates were merged using SAMtools merge (V1.8, http://www.htslib.org/doc/samtools-merge.html) and that file was further processed to text file with bamToBed using bedtools (V2.22.1, https://bedtools.readthedocs.io/en/latest/). The processed files were also utilized to create ‘.hic’ format files with the juicer tool (Durand et al. 2016). The previously published zebrafish HiC data sets that were used in this paper were from (Kaaij et al. 2018) (GSE105013). The valid pairs from the supplementary file provided on Geo were used for supplemental figure 2 and supplemental table 1.

**A/B compartment detection**

A/B compartments were called utilizing HiCExplorer (V2.1.4, https://hicexplorer.readthedocs.io/en/latest/content/News.html#release-2-1-4) with hicPCA on 100kb normalized and corrected matrices for each timepoint to create the BigWig of the first and second principal component analysis. hicTransform option –method all, created the Pearson matrix that was used to plot regions of interest along with the pca1.bw in hicPlotMatrix.

**Insulation score and boundary calling**

Domains and domain boundaries were identified by hicFindTADs command HiCExplorer (V3.3). The TAD separation scores in embryos were first calculated for a range of threshold comparisons from 0.001-0.5 (sup Fig 3A). Ultimately, boundaries were calculated with the following both N=10kb or 25kb resolution --step N --thresholdComparisons 0.05 --delta 0.01 --correctForMultipleTesting Bonferroni.

To remove false positive boundary calls due to genome assembly issues we visually inspected the boundaries called in the 2.25hpf (preZGA) and the 4hpf timepoints, where the least structure is present, for both 10kb and 25kb boundaries and determined a list of blacklisted regions. These regions were intersected and removed from all boundaries called for each timepoint utilizing a 30kb (for 10kb resolution) and 130kb (for 25kb resolution) window. Domains were identified as regions between two boundaries, after removing blacklisted boundaries, and no more than 500kb distance between two boundaries as done in (Hug et al. 2017).

To map insulation score using deeptools (V2.0, <https://deeptools.readthedocs.io/en/develop/content/list_of_tools.html>). The bedGraph of ‘score.bedGraph’ file in hicFindTADs program was converted to a BigWig with bedGraphToBigWig. This BigWig was then used in plotting insulation score of all samples with a given bed file.

**Flare/hinge calling**

Flare/hinge regions in sperm Hi-C data was found by extracting the positive values from the last column of the bedGraph matrix in the ‘tad_score.bm’ file from hicFindTADs command HiCExplorer (V3.3). Flares/hinges were merged if within 50kb of each other and the first round of filtering was done to remove blacklisted regions as described previously. A second round of filtering was done by visually inspecting the positive flares and verifying they were not a false positive due to a genome assembly issue. Once flare/hinge list was filtered, the size of a flare was calculated by the width of the positive values in the bedGraph matrix. The distance between two flares was calculated by measuring the distance of one flare to the other. The distance between flares was excluded if there was a genome assembly gap creating a blacklisted region causing an inaccurate distance measurement.

**GC fractionation and TSS enrichment analysis**

GC fractionation over each Hinge like domains as determined by CpG_calculator.pl (Biotoolbox, https://github.com/tjparnell/biotoolbox), as compared to a shuffled region of the genome. The GC fraction for each Hinge was mapped in Prism 8 as a violin plot. Odds ratios and p values were calculated using Prism 8 using Fisher’s exact test on a contingency table of TSS versus not TSS genes and Flare versus no Flare with Bonferroni correction. The list of genes that are expressed as firstwave, ZGA, 5.3hpf TSS’s were obtained from (Chan et al. 2019). For all TSS’s regardless of expression were found through UCSC Genome browser tables.

**Simulated Hi-C data processing**

The 2.25hpf -early dechorionated (preZGA), 24hpf, and sperm were aligned to the Zv10 genome alone. HiCExplorer (V3.3, https://hicexplorer.readthedocs.io/en/latest/) hicBuildMatrix was used to create matrix at 25kb resolutions, using the option –outBam (To extract valid Hi-C reads). The validpairs bam files for 24hpf and sperm were subsampled, utilizing picard.jar DownsampleSam (https://gatk.broadinstitute.org/hc/en-us/articles/360037056792-DownsampleSam-Picard-), to 100, 50, 40, 30, 20 million reads. The preZGA validpairs bam file was subsampled to 100, 50, 60, 70, 80. The downsampled bam files were then split to FASTQ files and realigned to the Zv10 genome. Hi-C Matrices were made again as described above and the appropriately mixed matrices were summed utilizing hicSumMatrices (50:50, 60:40, 70:30, 80:20) and were corrected prior to visualizing with hicPlotMatrix. TADs were called for each simulated Hi-C matrix, utilizing the above specifications, to measure the insulation score genome wide. The analysis of the insulation score centered on the boundaries called in the 24hpf sample was described as above. The metaplots were visualized in R (V3.3.3, 2017) (Team 2017) utilizing ggplot2

**ChIP-seq data processing and peak calling.**

Fastq files were aligned to Zv10 using novoalign (version 2.8) with the following parameters: -r Random -o SAM. SAM files were converted to BAM format and sorted with SAMtools (V1.8, http://www.htslib.org/doc/samtools). BAM files for technical replicates were merged using SAMtools merge (V1.8, http://www.htslib.org/doc/samtools-merge.html). Paired-end ChIP-seq data with multiple biological replicates and their corresponding input data were then processed using Multi-Replica Macs ChIPSeq Wrapper (https://github.com/HuntsmanCancerInstitute/MultiRepMacsChIPSeq) with the following parameters: --species zebrafish --pe --nodup --cutoff 2 --tdep N, N represents the smallest read counts among replicates divided by 10^6^. The log_2_ fold enrichment BigWig files were used for generating heatmaps and metaplots. For single-end ChIP-seq, fragment sizes were checked using Macs2 predicted and bam2wig.pl (Biotoolbox, https://github.com/tjparnell/biotoolbox), for IP and corresponding input bam files. The MultiRepMacsChIPSeq (version 9, https://github.com/HuntsmanCancerInstitute/MultiRepMacsChIPSeq) with options: --species zebrafish --size M --nodup --cutoff 2 --tdep N, M represents the predicted fragment size. For published ChIP-seq without biological replicates and input, peaks were called using Macs2 callpeak -B --fix-bimodal --extsize 250 -q 0.01 --SPMR. Fold enrichment was generated from bedGraph files of treat_pileup and control_lambda with Macs2 bdgcmp, which then was converted to log_2_ fold enrichment by convert_FE_to_log2FE.pl script (Biotoolbox, <https://github.com/tjparnell/biotoolbox>). The available ChIP-seq data sets that were used in this paper were as followed: Sperm ChIP seq H3K27ac H3K27me3 and H3K4me3 (GSM3165194); 24hpf histone H3K4me3 (GSE71434), histone H3K27me3 (GSE35050) histone H3K27ac (GSE32483); 4hpf histone H3K27ac (GSE114954), histone H3K27me3 (GSE114954), histone H3K36me3(GSE114954), histone H3K4me3 (GSE114954), histone H3K4me1(GSE32483).

**ATAC-seq data processing**

Fastq files were aligned to Zv10 using novoalign with the following parameters: -r Random -o SAM. SAM files were converted to BAM format and sorted with SAMtools (V1.8, http://www.htslib.org/doc/samtools). BAM files for technical replicates were merged using SAMtools merge (V1.8, http://www.htslib.org/doc/samtools-merge.html). Data with multiple biological replicates were then processed using Multi-Replica Macs ChIPSeq Wrapper(https://github.com/HuntsmanCancerInstitute/MultiRepMacsChIPSeq) with the following parameters: --species zebrafish --pe --min 30 --max 120--nodup --cutoff 2 --tdep N, N represents the smallest read counts among replicates divided by 10^6. The fragment sizes were set to be subnucleosomal to allow for identification of transcription factor binding. The log_2_ fold enrichment BigWig files were used for generating heatmaps and metaplots.

**Click-iT-seq and RNA-seq data processing**

The published Click-iT-seq data set available from (SRP184786) files were aligned to Zv10 genome using STAR 2.5.3a with the following parameters: --twopassMode Basic --quantMode TranscriptomeSAM --outFilterMultimapNmax 100 --winAnchorMultimapNmax 200 --outMultimapperOrder Random --outSAMmultNmax 1 --outWigType bedGraph. Bedgraph files were converted to BigW­ig files with UCSC bedGraphToBigWig.

The published RNA-seq TPM were downloaded from Expression Atlas E-ERAD-475. The z-score and heatmap were created in R (R: A language and Environment for Statistical Computing, R Core Team, R Foundation for Statistical Computing, Vienna, Austria, 2017, <https://www.R-project.org>), using ComplexHeatmap. Raw gene counts were obtained from (Expression Atlas E-ERAD-475). Differentially expressed genes were identified by DESeq2 (q<0.01, fold change>=1.5).

**ROSE enhancer algorithm and sequence motif enrichment analysis**

To identify enhancers and super enhancers (SE) the ROSE algorithm version 0.1 was applied with default parameters performing TSS exclusion (–t 2000) (Loven et al. 2013; Whyte et al. 2013). Using the intersected peaks between H3K4me1 and H3K27ac ChIP-seq signal (this list also excluded promoters). For the 4hpf enhancers, were stratified into three equal sized cohorts for further examination while the super enhancers remained at N=411. For the 24hpf potential enhancers, the enhancers list in (Perez-Rico et al. 2017) were lifted over using UCSC to ZV10 and utilized in the ROSE algorithm version 0.1. Candidate transcription factor motifs was determined by intersecting potential enhancers with ATAC-seq narrowpeaks signal and this list was used in HOMER findMotifsGenome.pl to find known binding motifs and motif frequency. The list was cross-referenced with Click-iT-seq data to verify expression, regardless of maternal contribution, of potential transcription factor in the early embryo. Bubble plot was created in R (R: A language and Environment for Statistical Computing, R Core Team, R Foundation for Statistical Computing, Vienna, Austria, 2017, <https://www.R-project.org>), using standard methods. CTCF motifs were determined by HOMER findMotifsGenome.pl, potential CTCF locations were determined by converting the HOMER output to wig files and ran in Danpos (Chen et al. 2013) for locations.

**Metaregion analysis for HiC insulation, ChIP-seq, ATAC-seq, and Click-iT-seq**

To generate metaregions plots of ChIP-seq, ATAC-seq, and Click-iT-seq signal was averaged in 10kb bins across the genome using get_datasets.pl from Biotoolbox (https://github.com/tjparnell/biotoolbox). To generate metaregion plots for HiC insulation the bedGraph matrix in the ‘tad_score.bm’ file from hicFindTADs command HiCExplorer (V3.3) was converted to a bigWig using UCSC bedGraphToBigWig. These resulting files were used to make heatmaps across either boundaries, enhancers, or flares generated with deeptools (V2.0, <https://deeptools.readthedocs.io/en/develop/content/list_of_tools.html>). The metaplots were visualized in R (V3.3.3, 2017) using standard methods.


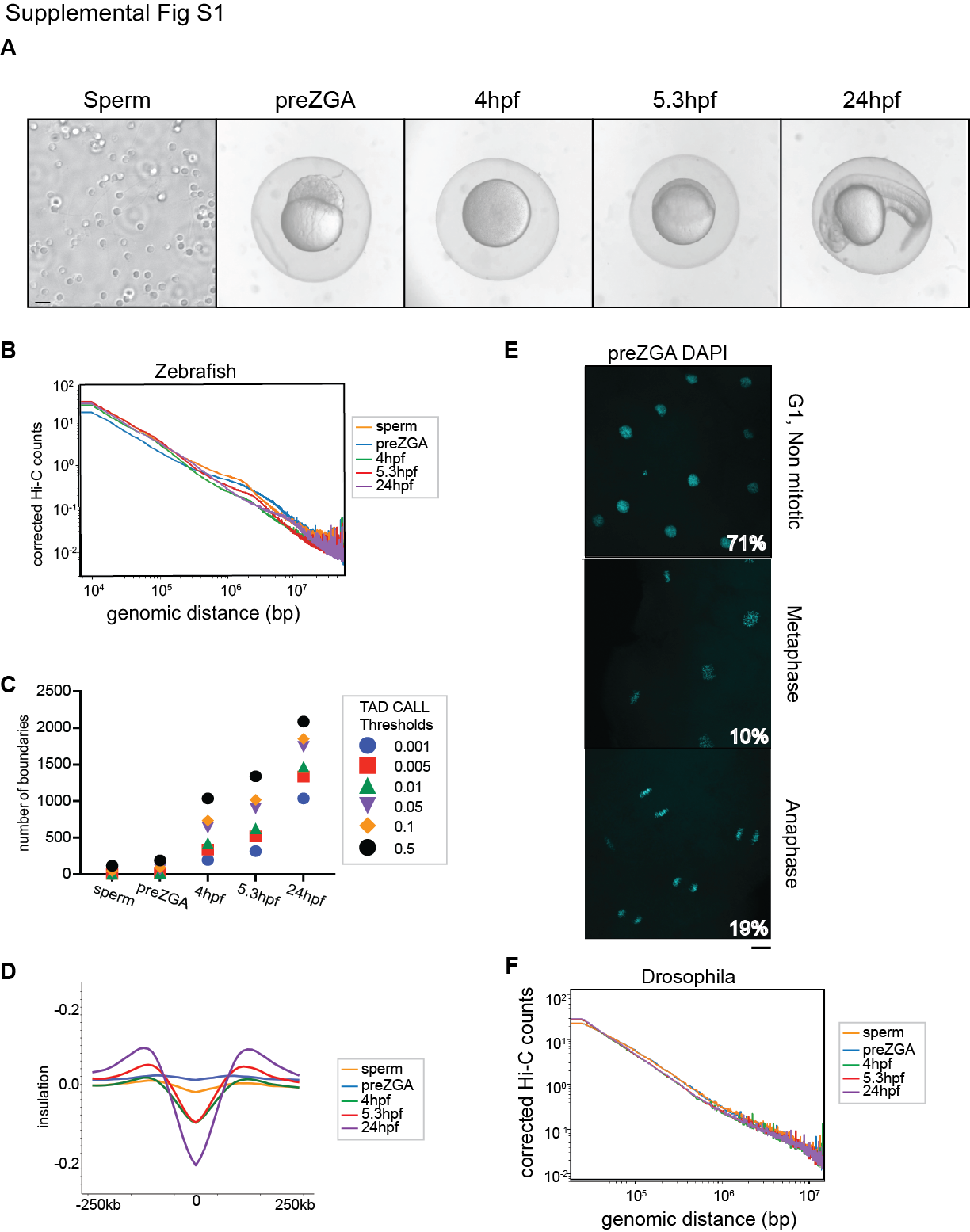


Supplemental Fig S1. Quality control analysis of zebrafish embryos and contact maps.

1. Representative brightfield images of sperm, and embryos collected at the indicated timepoints.
2. Zebrafish contact probability plotted against genomic distance the combined contact maps from each developmental timepoint collected are shown.
3. Number of TAD-boundary calls for different boundary thresholds at each developmental time point.
4. Metaplot of the insulation of each developmental timepoint Sperm (orange), preZGA (blue), 4hpf (green), 5.3hpf (red), 24hpf (purple) centered on boundaries determined at 24hpf (threshold 0.05, 10kb resolution).
5. Fluorescent microscopy images of DAPI-stained embryos at preZGA (2.25hrs). Signal from DAPI reveals the cell stage synchronization of the embryos. The percentage of embryos in each cell cycle stage collected in the preZGA sample used in Figure 1 is marked in the bottom right of each image.
6. Contact probability for the *Drosophila* spike in, plotted against genomic distance the combined contact maps from each developmental timepoint collected are shown.


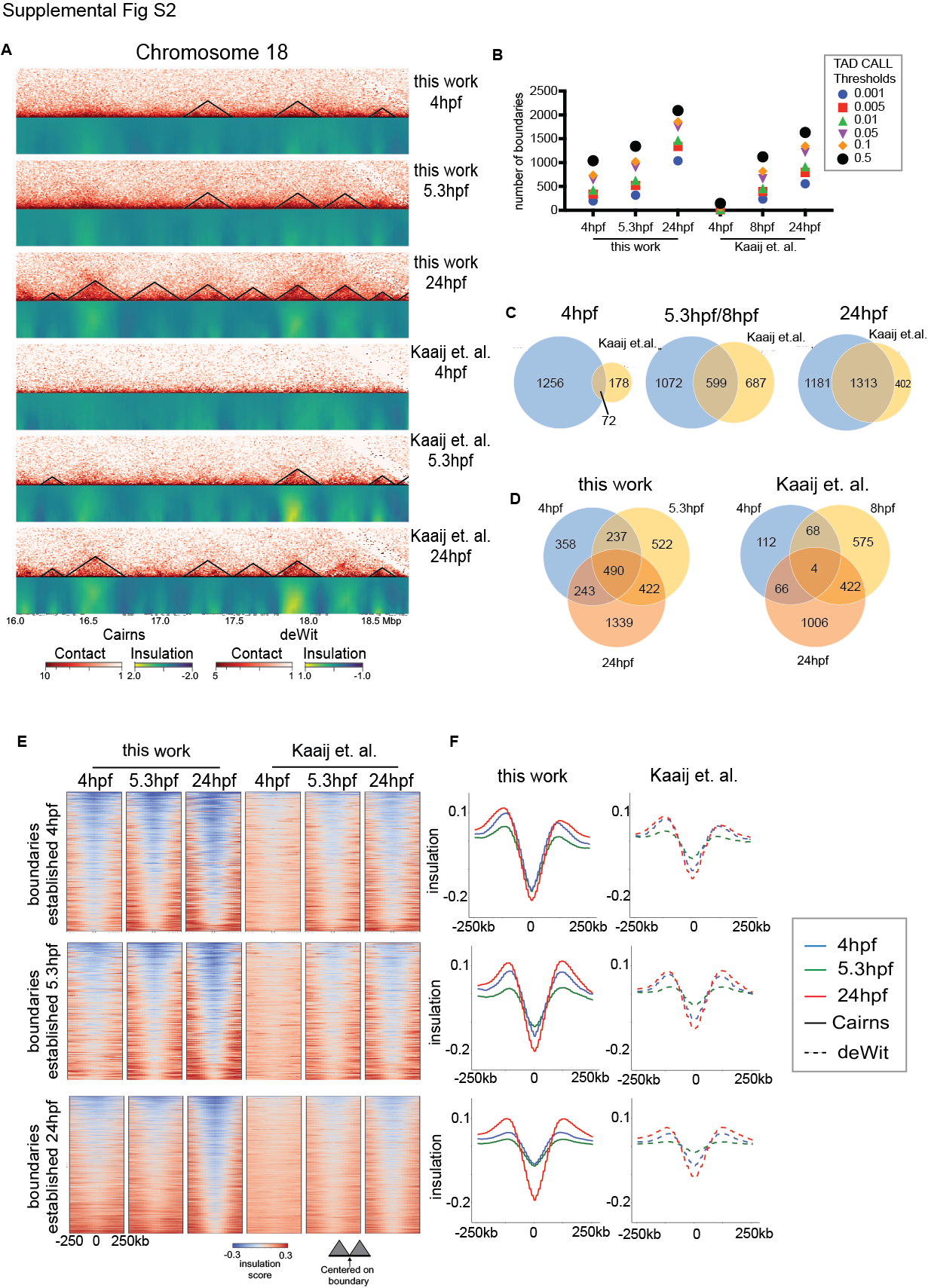


Supplemental Fig. S2 Establishment of boundaries during zygotic genome activation.

1. Hi-C contact maps (10kb resolution in log scale) and Heatmap of insulation scores at different window sizes for 4hpf, 5.3hpf and 24hpf for this work and previously published (Kaaij et al. 2018) samples. Black lines overlaid on contact maps depict detectable domains and boundaries for each timepoint.
2. Number of TAD-boundary calls for different boundary thresholds at each developmental time point for this work, and the previously published (Kaaij et al. 2018) samples.
3. Venn diagram indicate overlap of called boundaries comparing this work (blue) to Kaaij et al., 2018 (yellow) for 4hpf (left), 5.3hpf (middle) and 24hpf (right) samples.
4. Venn diagram indicate overlap of called boundaries comparing between each timepoint 4hpf (blue) 5.3hpf (yellow), and 24hpf (orange) for this work (left) and Kaaij et al., 2018 samples.
5. Heatmaps of insulation scores at newly established boundaries for 4hpf (top) 5.3hpf (middle) and 24hpf (bottom). Positive insulation (red) indicates increased contacts and negative insulation (blue) indicates a lack of contacts.
6. Insulation score at established boundaries averaged across regions depicted in (E) for this work (left, solid line) and Kaaij et al., 2018 (right, dashed line) samples.


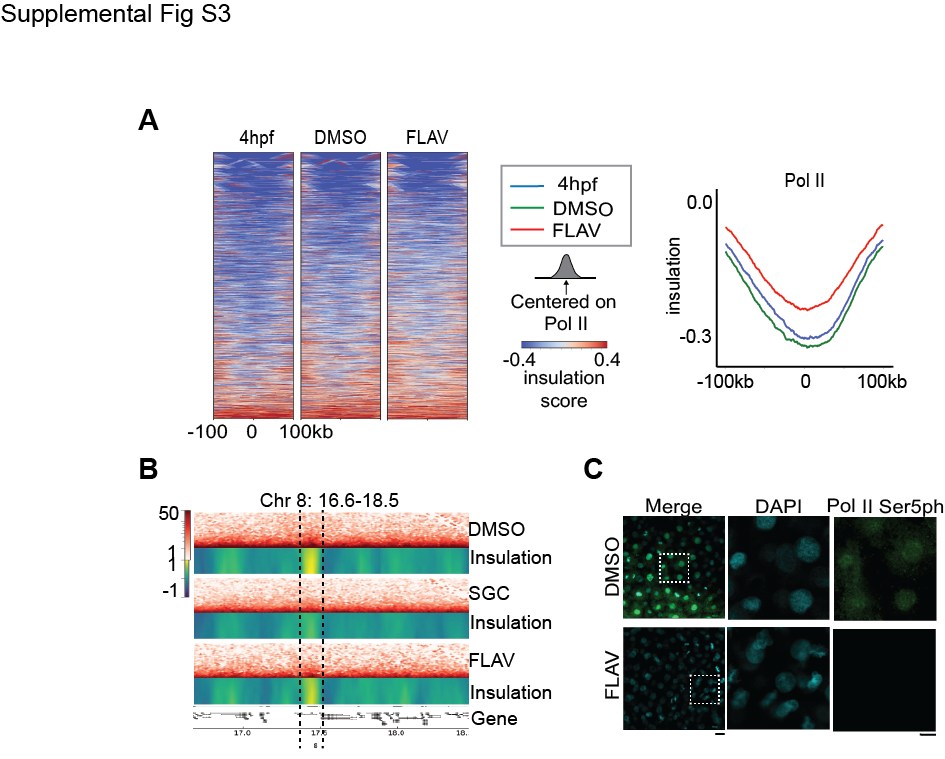


Supplemental Fig. S3 Dynamics of chromatin architecture at RNA Polymerase II binding sites upon transcriptional inhibition

1. Insulation score at the top 1000 Polymerase (Pol II) binding sites based on ChIP-seq at 4hpf (left), vehicle control (DMSO, middle) and flavopiridol (FLAV, right). Positive insulation (red) indicates increased contacts and negative insulation (blue) indicates a lack of contacts. The metaplot of insulation across the top 1000 Pol II binding sites is plotted (right) for 4hpf (blue) DMSO (green) and FLAV (red).
2. Representative contact maps for DMSO SGC and FLAV treated samples each presented for a 2Mb region on Chr 8 at 25kb resolution in log scale (top). Heatmap of insulation scores for different window sizes (bottom). Positive insulation (yellow) indicates increased contacts and negative insulation (blue) indicates a lack of contacts. The gene locations and distance are mapped
3. Immunofluorescence of Pol II ser5 (green) upon DMSO (top) FLAV (bottom) treatment. DNA is stained with DAPI. The white box indicates magnified area. Scale bar is 20um for merged panel and 10uM for DAPI and Pol II Ser5ph.


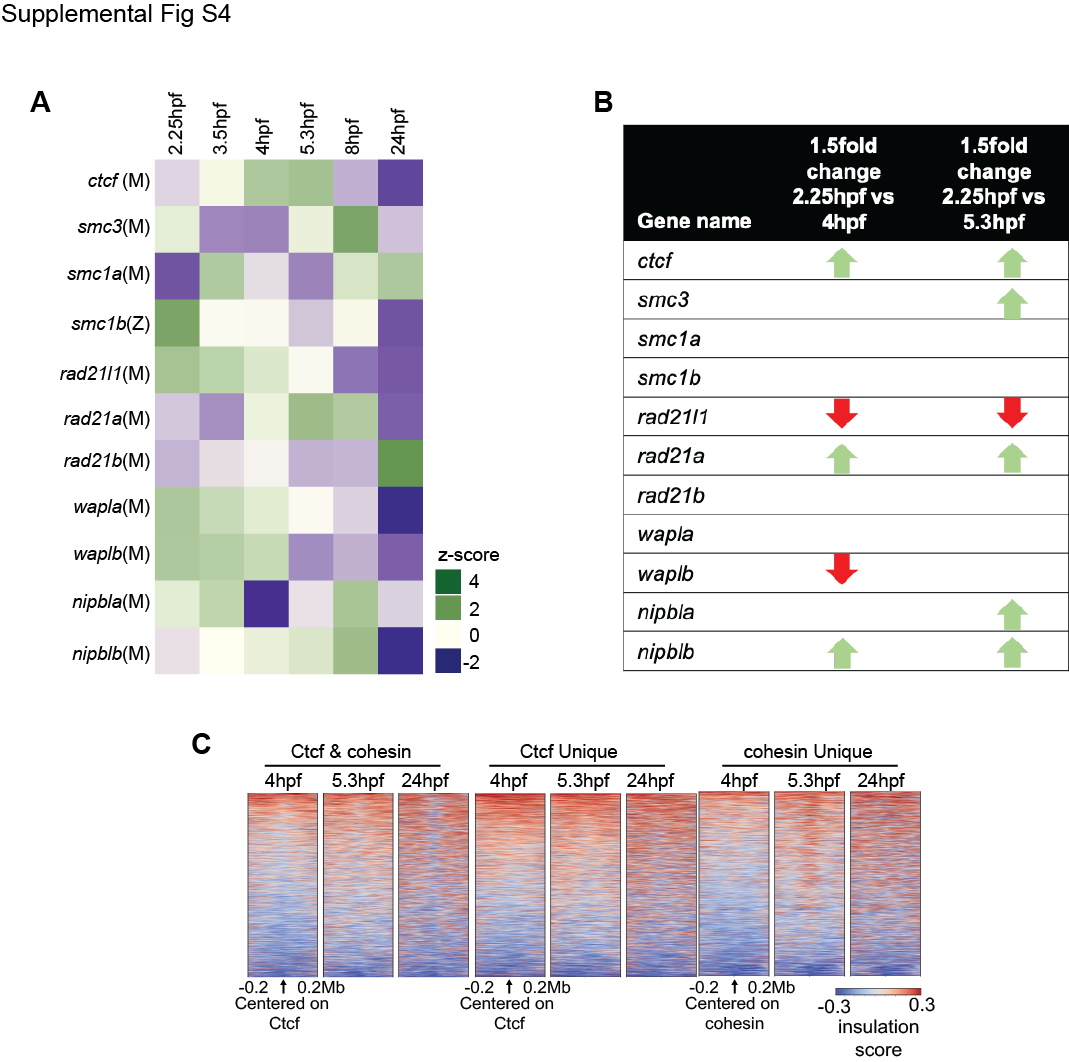


Supplemental Fig. S4 Chromatin architecture insulation at Ctcf motif and cohesin binding sites.

1. Heatmap of RNA-Seq expression z-scores computed for all genes through each column in the heatmap corresponds to the developmental times 2.25hpf, 3.5hpf, 4hpf, 5.3hpf, 8hpf, and 24hpf. Each row corresponds to a specific expressed gene as labeled. The genes are marked with (M) for maternal contribution or (Z) for Zygotic contribution as determined by Click-iT-seq from (Chan et al. 2019). The RNA-seq counts were obtained from (White et al. 2017).
2. Chart demonstrating the genes that are changing by 1.5 fold differential expression of genes between 2.25hpf and 4hpf (left) or 2.25 and 5.3hpf (right), as determined by DEseq2 in R package. The RNA-seq counts were obtained from (White et al. 2017). Green arrow means gene expression is up significantly and red arrow means gene expression is down significantly by 1.5 fold.
3. Heatmaps of insulation, centered on regions co-occupied by Ctcf binding sites and Rad21/cohesin (left), regions bearing Ctcf motifs but not Rad21/cohesin (middle) and regions (at 4hpf) occupied by cohesin but not Ctcf binding sites, at each developmental timepoint 4hpf, 5.3hpf, and 24hpf. Deeptools heatmap ranked by insulation. Positive insulation (red) indicates increased contacts and negative insulation (blue) indicates a lack of contacts.


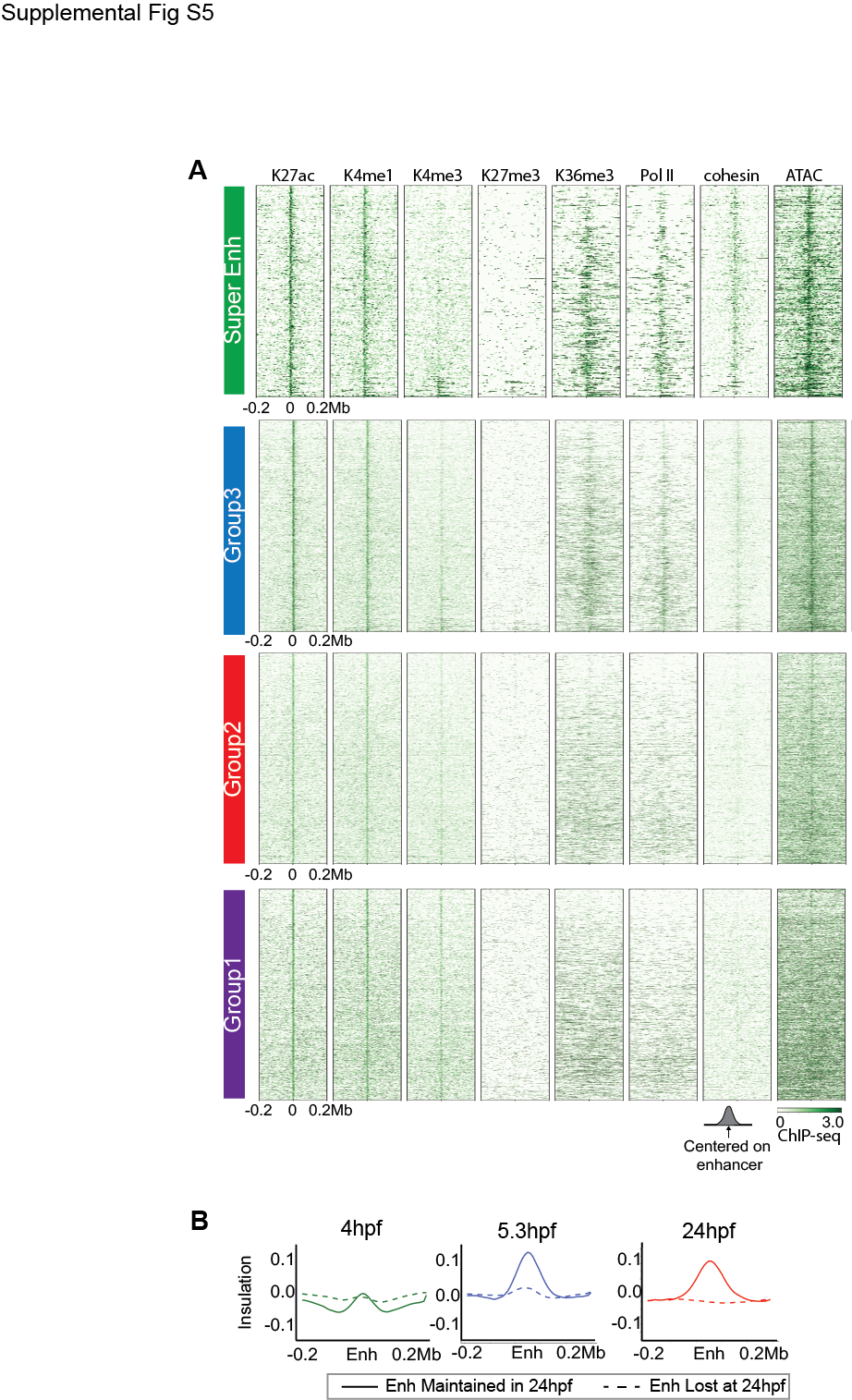


Supplemental Fig. S5 Chromatin characteristics at enhancers during zygotic genome activation.

1. Heatmaps centered on enhancers of each respective enhancer group ranked as in Figure 3B. Log2 fold enrichment of histone H3K27ac (Zhang et al. 2018), H3K4me1 (Bogdanovic et al. 2012), H4K3me3 (Zhang et al. 2016), H3K27me3 (Zhang et al. 2018), H3K36me3 (Zhang et al. 2018), Pol II, and cohesin ChIP-seq signal over input and ATAC-seq for 4hpf embryo.
2. Metaplot of the insulation score for 4hpf (green), 5.3hpf (blue), and 24hpf (red) centered on enhancers (Enh) with 200kb upstream and downstream. The distinguishing lines depict if the enhancers called at 4hpf is maintained at 24hpf (solid) or lost at 24hpf (dashed).


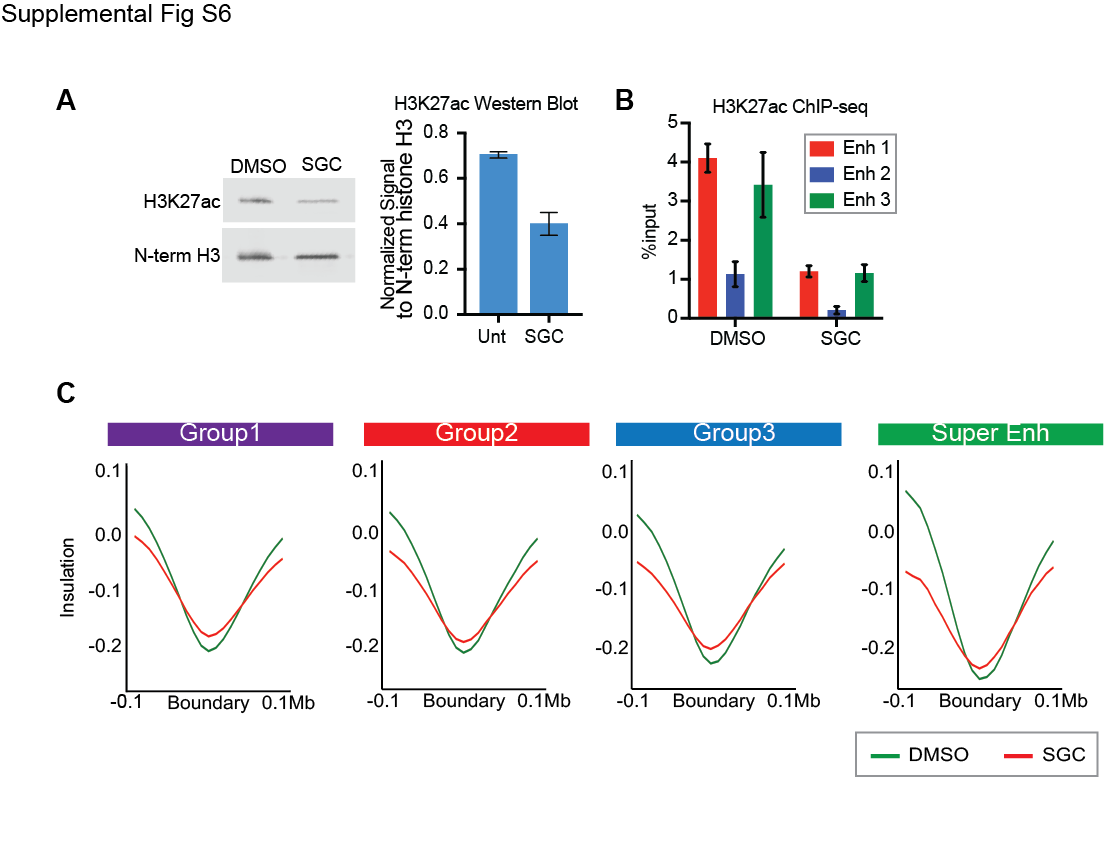
Supplemental Fig. S6 Boundary insulation in the early zebrafish embryo remains relatively unchanged following treatment with the inhibitor SGC.

1. Zebrafish embryo extracts collected at 4hpf following treatment with vehicle (DMSO) or SGC-CBP30 (SGC) were immunoblotted, and probed with antibodies raised against H3K27ac or the N-terminus of Histone H3 (left). The western/immunoblot is a representative image from three different collections. Protein quantification based on band intensities from the immunobot using the LiCor system (right). The amount of H3K27ac was normalized to the signal derived from the anti-N-terminal Histone H3 band. Error bars represent standard deviation of three experiments.
2. Levels of histone H3K27ac ChIP-qPCR present at three different enhancer regions called by ROSE, and normalized to input control, for DMSO and SGC treated embryos
3. Metaplot of the insulation at boundaries derived from 4hpf embryos, following treatment by DMSO (green) or SGC (red), centered on the boundary closest to the enhancer of each respective group, with 100kb upstream and downstream depicted.


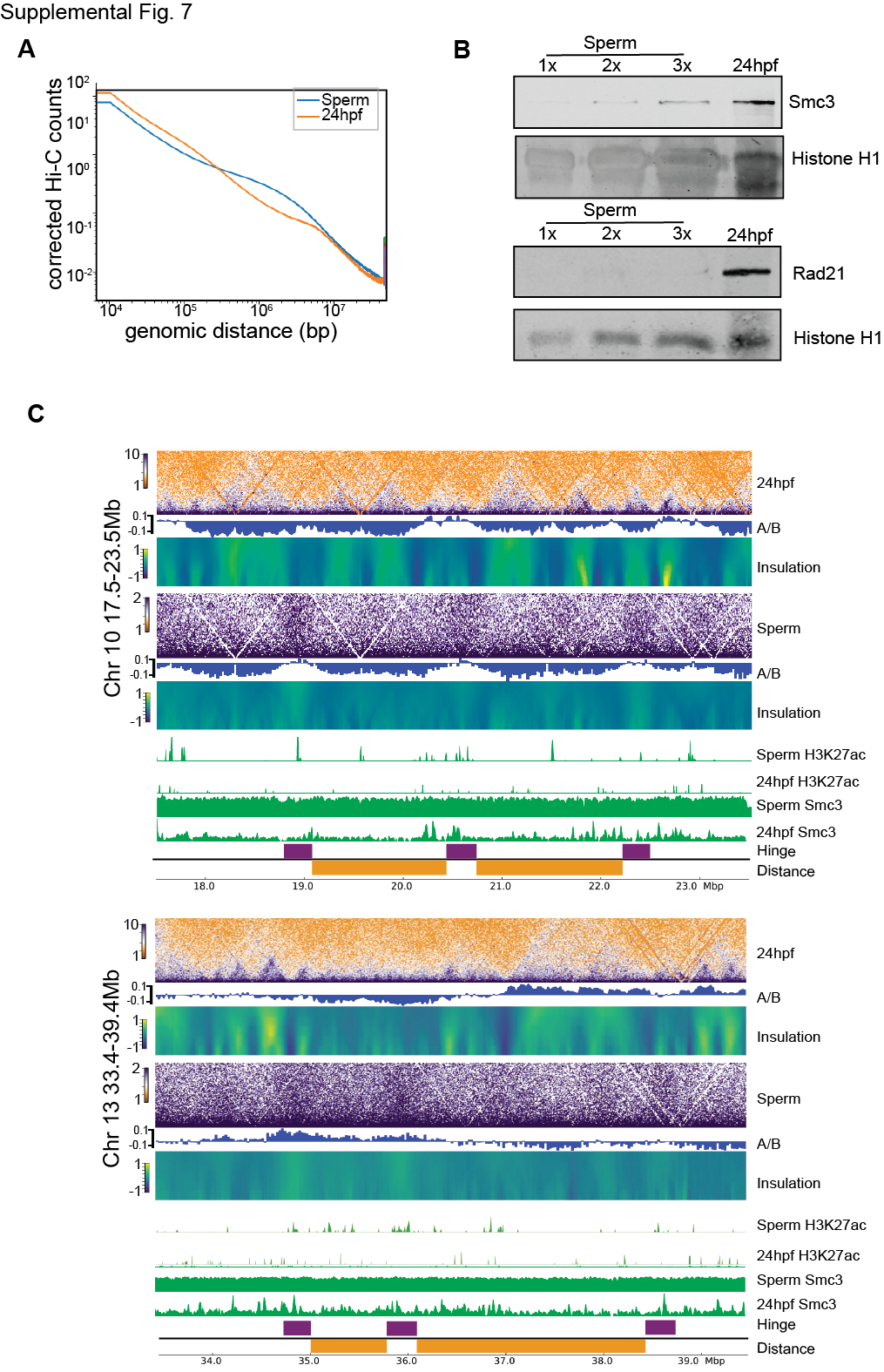
Supplemental Fig. S7 No discrete peaks are detected in sperm for Smc3

1. Contact probability plotted against sperm and 24hpf from Fig. S1B.
2. Zebrafish sperm and 24hpf embryo extracts were probed with Smc3 (top) and Rad21 (bottom), and Histone H1 (loading control). Sperm was loaded in increasing concentrations (1x, 2x, 3x). 24hpf is loaded equivalent to the 2x concentration.
3. Two additional examples of contact maps for 24hpf and sperm samples each presented for a 6Mb region on Chr 10 and 13 each is at 25kb resolution in log scale (top). First principal component values to determine A/B compartment status (middle). Heatmap of insulation scores for different window sizes (bottom). Genome browser snapshots of the occupied regions for histone H3K27ac (Zhang et al. 2018) and Smc3 in sperm and 24hpf timepoints (green). The hinge region is marked by purple square and distance is marked by orange square.


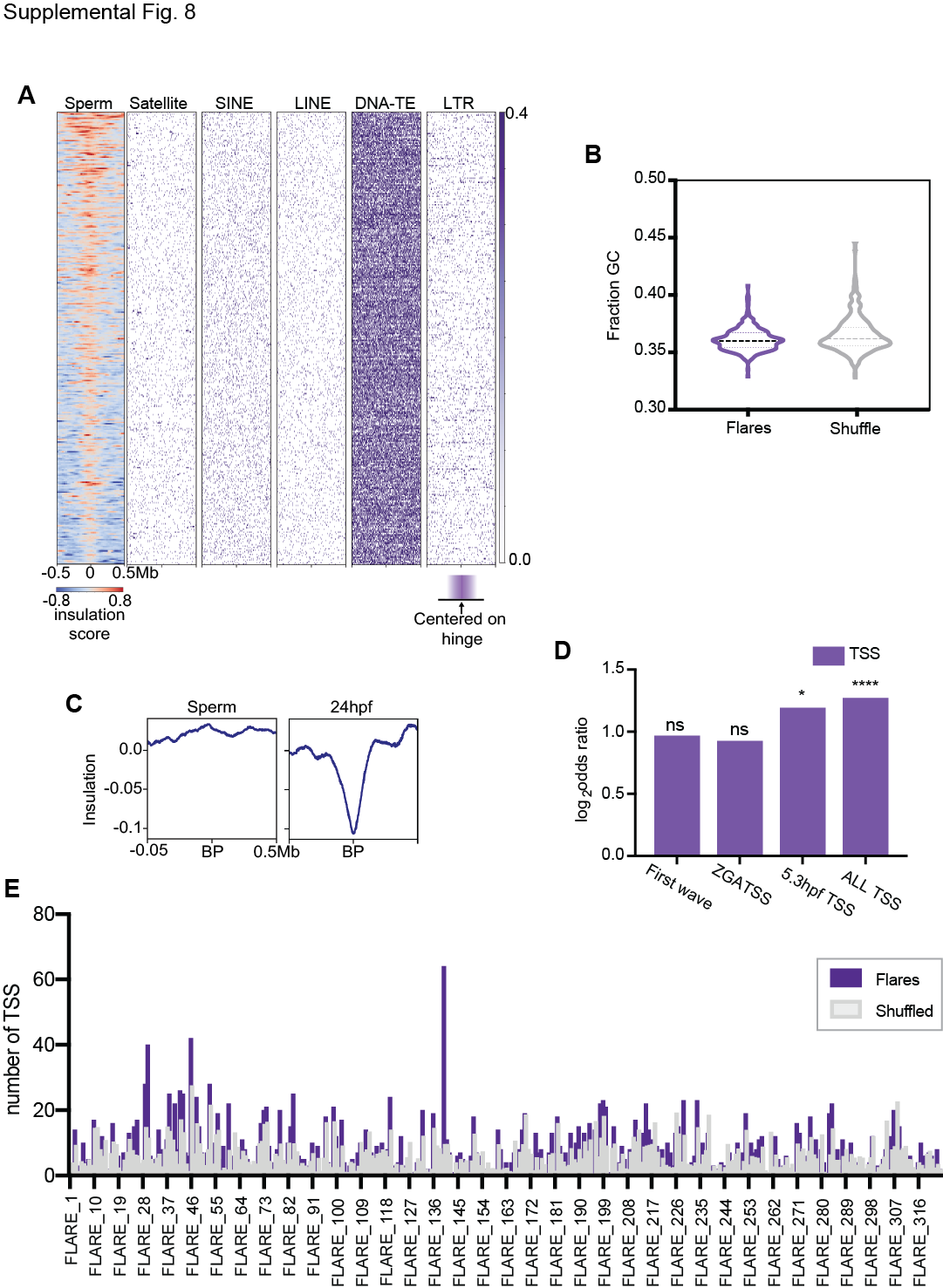


Supplemental Fig. S8 Genomic features at Hinge-Like Domains

(A) Heatmaps of repetitive elements at hinge like domains. The left is the Insulation map of sperm ranked by insulation strength, centered on hinge-like domains from Figure 6C. Positive insulation (red) indicates increased contacts and negative insulation (blue) indicates a lack of contacts. The right is the heatmaps centered on hinge for counts of different elements in the genome: Satellite, SINE, LINE, DNA-TE, LTR, and tRNA (marked in purple).

(B) GC fractionation over each Hinge like domains as compared to a shuffled region of the genome. The GC fraction for each Hinge was mapped as a violin plot.

(C) Insulation across breakpoints in zebrafish. Breakpoints were obtained from (Yang et al. 2020) and the metaplot of the insulation of sperm or 24hpf centered on the breakpoint is shown 500kb upstream and downstream.

(D) Odds ratio of transcription start sites (TSS) within a hinge. The list of genes that are

expressed as firstwave, ZGA, 5.3hpf TSS’s were obtained from (Chan et al. 2019). For all TSS’s regardless of expression were found through UCSC Genome browser tables. Significance was determined by Fisher’s Exact test p-value (n.s. no significance, **** p=0.0001).

(E) The bar chart demonstrates counts all TSS (purple) found per flare/hinge compared to a shuffled list (grey) of random genome locations. The higher number of TSS in Flare 138 is due to one gene repeat cluster and is not of functional significance.

**Supplemental References**

Bogdanovic O, Fernandez-Minan A, Tena JJ, de la Calle-Mustienes E, Hidalgo C, van Kruysbergen I, van Heeringen SJ, Veenstra GJ, Gomez-Skarmeta JL. 2012. Dynamics of enhancer chromatin signatures mark the transition from pluripotency to cell specification during embryogenesis. *Genome Res* **22**: 2043-2053.

Buenrostro JD, Wu B, Chang HY, Greenleaf WJ. 2015. ATAC-seq: A Method for Assaying Chromatin Accessibility Genome-Wide. *Curr Protoc Mol Biol* **109**: 21 29 21-21 29 29.

Chan SH, Tang Y, Miao L, Darwich-Codore H, Vejnar CE, Beaudoin JD, Musaev D, Fernandez JP, Benitez MDJ, Bazzini AA et al. 2019. Brd4 and P300 Confer Transcriptional Competency during Zygotic Genome Activation. *Dev Cell* **49**: 867-881 e868.

Chen K, Xi Y, Pan X, Li Z, Kaestner K, Tyler J, Dent S, He X, Li W. 2013. DANPOS: dynamic analysis of nucleosome position and occupancy by sequencing. *Genome Res* **23**: 341-351.

Diaz N, Kruse K, Erdmann T, Staiger AM, Ott G, Lenz G, Vaquerizas JM. 2018. Chromatin conformation analysis of primary patient tissue using a low input Hi-C method. *Nat Commun* **9**: 4938.

Durand NC, Shamim MS, Machol I, Rao SS, Huntley MH, Lander ES, Aiden EL. 2016. Juicer Provides a One-Click System for Analyzing Loop-Resolution Hi-C Experiments. *Cell Syst* **3**: 95-98.

Goren A, Ozsolak F, Shoresh N, Ku M, Adli M, Hart C, Gymrek M, Zuk O, Regev A, Milos PM et al. 2010. Chromatin profiling by directly sequencing small quantities of immunoprecipitated DNA. *Nat Methods* **7**: 47-49.

Hagege H, Klous P, Braem C, Splinter E, Dekker J, Cathala G, de Laat W, Forne T. 2007. Quantitative analysis of chromosome conformation capture assays (3C-qPCR). *Nat Protoc* **2**: 1722-1733.

Hug CB, Grimaldi AG, Kruse K, Vaquerizas JM. 2017. Chromatin Architecture Emerges during Zygotic Genome Activation Independent of Transcription. *Cell* **169**: 216-228 e219.

Kaaij LJT, van der Weide RH, Ketting RF, de Wit E. 2018. Systemic Loss and Gain of Chromatin Architecture throughout Zebrafish Development. *Cell Rep* **24**: 1-10 e14.

Kroeger PT, Jr., Poureetezadi SJ, McKee R, Jou J, Miceli R, Wingert RA. 2014. Production of haploid zebrafish embryos by in vitro fertilization. *J Vis Exp* doi:10.3791/51708.

Loven J, Hoke HA, Lin CY, Lau A, Orlando DA, Vakoc CR, Bradner JE, Lee TI, Young RA. 2013. Selective inhibition of tumor oncogenes by disruption of super-enhancers. *Cell* **153**: 320-334.

Perez-Rico YA, Boeva V, Mallory AC, Bitetti A, Majello S, Barillot E, Shkumatava A. 2017. Comparative analyses of super-enhancers reveal conserved elements in vertebrate genomes. *Genome Res* **27**: 259-268.

Picelli S, Bjorklund AK, Reinius B, Sagasser S, Winberg G, Sandberg R. 2014. Tn5 transposase and tagmentation procedures for massively scaled sequencing projects. *Genome Res* **24**: 2033-2040.

Rao SS, Huntley MH, Durand NC, Stamenova EK, Bochkov ID, Robinson JT, Sanborn AL, Machol I, Omer AD, Lander ES et al. 2014. A 3D map of the human genome at kilobase resolution reveals principles of chromatin looping. *Cell* **159**: 1665-1680.

Team RC. 2017. R: A Language and Environment for Statistical Computing

. *R Foundation for Statistical Computing*.

White RJ, Collins JE, Sealy IM, Wali N, Dooley CM, Digby Z, Stemple DL, Murphy DN, Billis K, Hourlier T et al. 2017. A high-resolution mRNA expression time course of embryonic development in zebrafish. *Elife* **6**.

Whyte WA, Orlando DA, Hnisz D, Abraham BJ, Lin CY, Kagey MH, Rahl PB, Lee TI, Young RA. 2013. Master transcription factors and mediator establish super-enhancers at key cell identity genes. *Cell* **153**: 307-319.

Yang H, Luan Y, Liu T, Lee HJ, Fang L, Wang Y, Wang X, Zhang B, Jin Q, Ang KC et al. 2020. A map of cis-regulatory elements and 3D genome structures in zebrafish. *Nature* **588**: 337-343.

Zhang B, Wu X, Zhang W, Shen W, Sun Q, Liu K, Zhang Y, Wang Q, Li Y, Meng A et al. 2018. Widespread Enhancer Dememorization and Promoter Priming during Parental-to-Zygotic Transition. *Mol Cell* **72**: 673-686 e676.

Zhang B, Zheng H, Huang B, Li W, Xiang Y, Peng X, Ming J, Wu X, Zhang Y, Xu Q et al. 2016. Allelic reprogramming of the histone modification H3K4me3 in early mammalian development. *Nature* **537**: 553-557.
